# Structural Considerations for Building Synthetic Glycoconjugates as Inhibitors for *Pseudomonas aeruginosa* Lectins

**DOI:** 10.1002/cmdc.202200081

**Published:** 2022-05-03

**Authors:** Karolina Wojtczak, Joseph P. Byrne

**Affiliations:** ^1^ School of Biological and Chemical Sciences National University of Ireland Galway University Road Galway Ireland

**Keywords:** carbohydrate-protein interactions, glycoconjugates, lectins, *Pseudomonas aeruginosa*, biofilms

## Abstract

*Pseudomonas aeruginosa* is a pathogenic bacterium, responsible for a large portion of nosocomial infections globally and designated as critical priority by the World Health Organisation. Its characteristic carbohydrate‐binding proteins LecA and LecB, which play a role in biofilm‐formation and lung‐infection, can be targeted by glycoconjugates. Here we review the wide range of inhibitors for these proteins (136 references), highlighting structural features and which impact binding affinity and/or therapeutic effects, including carbohydrate selection; linker length and rigidity; and scaffold topology, particularly for multivalent candidates. We also discuss emerging therapeutic strategies, which build on targeting of LecA and LecB, such as anti‐biofilm activity, anti‐adhesion and drug‐delivery, with promising prospects for medicinal chemistry.

## Graphical abstract

**Structural features** which impact the binding affinity of glycoconjugates for LecA and LecB, the carbohydrate‐binding proteins produced by the pathogen *P. aeruginosa*, are explored in this review article. We also highlight promising therapeutic lectin‐targeting applications.

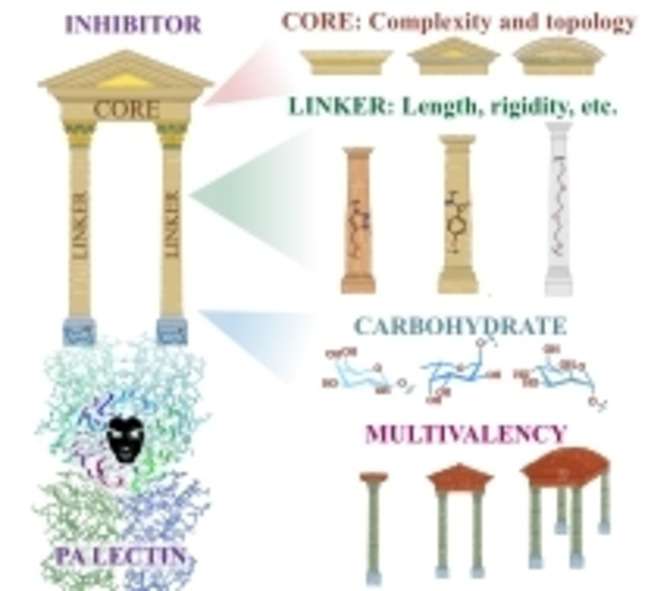

## 1 Introduction

*Pseudomonas aeruginosa* (PA) is a ubiquitous pathogenic bacteria, which is a leading cause of chronic infections and death among immunocompromised and hospitalised patients, including those with cystic fibrosis (CF).^[1]^ PA has been classified as a ‘Critical’ pathogen by the World Health Organisation and is of particular concern in light of the growing global problem of antimicrobial resistance.[^2^, ^3^, ^4^] Various approaches to treating PA, in addition to traditional antibiotics, have been reported including inhibition of quorum sensing, biofilm‐formation, iron‐chelation and interfering with biosynthetic pathways of the bacterium.[^5^, ^6^, ^7^] PA produces characteristic carbohydrate‐binding proteins, the soluble lectins LecA and LecB, which play a role in biofilm‐formation and lung‐infection.[^8^, ^9^, ^10^] An emerging anti‐PA strategy involves targeting these lectins with glycoconjugates. A vast array of monovalent and multivalent PA lectin inhibitors have been described, with structural variation having significant impact on binding affinity and selectivity, among other properties.

Many challenges still remain in designing effective, selective glycoconjugates for the purposes of lectin inhibition, which could be translated into clinical applications. This article will review the various synthetic strategies that have been employed in targeting these important pathogenic lectins in the last 15 years (>80 articles) and critically analyse beneficial or detrimental structural considerations. This comprehensive overview will be of value to a broad audience of synthetic and medicinal chemists in academia and in industry, aiming to build new therapeutic or diagnostic tools against PA. This topic is timely, and indeed some inspiring examples have been published in the last two years illustrating novel complementary therapeutic approaches, including targeted biofilm inhibition activity,^[11]^ directing antibiotics to the bacteria by lectin‐targeting,^[12]^ and hijacking the ability of the bacterium to utilise glycocluster‐conjugated siderophores for iron‐transport as a ‘Trojan horse’ strategy.^[13]^

### 1.1 Lectins LecA and LecB

**Figure 1:**
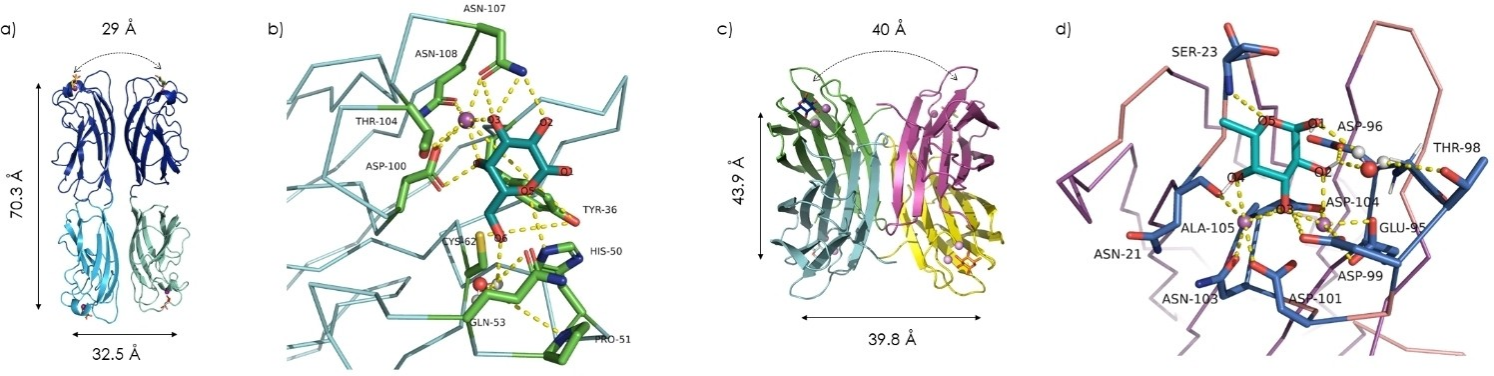
Structure of PA lectins: **a)** LecA tetramer structure (PDB 1OKO), with distances between Ca^2+^ (magenta spheres), and shortest distance between galactose‐binding sites (curved arrow) indicated; **b)** LecA binding‐site interactions with d‐Gal**; c)** LecB tetramer structure (PDB 1UZV), with distances between Ca^2+^, and shortest distance between fucose‐binding sites (curved arrow) indicated; **d**) LecB binding‐site interactions with l‐Fuc. (Images generated with PyMOL^[21]^).

LecA and LecB (formerly designated PA‐IL and PA‐IIL, respectively) have been well‐studied since their genetic origin[^14^, ^15^] and molecular structures were determined.^[8]^ They are expressed and released by PA as a result of a regulatory cascade initiated by quorum‐sensing.[^16^, ^17^] Upon release, they are involved in biofilm‐formation *via* recognition of bacterial envelope lipopolysaccharides, as well as mucins and cell surface‐glycans of the host, leading to adhesion and infection of tissues, particularly airway epithelial cells. LecA is demonstrated to be crucial for biofilm‐formation^[9]^ while LecB mediates interactions with exopolysaccharides.^[18]^ LecA also plays a key role in internalization of PA into host cells, as well as being cytotoxic, causing damage to lung‐ and gut‐epithelial cells. By contrast, LecB is not cytotoxic, but affects ciliary beating frequency of airway epithelial cells.^[19]^ Both are C‐type lectins dependent on the presence of Ca^2+^ and Mg^2+^ ions for their function and exist as tetramers in their natural form (Figure 1). The structural basis of their recognition of host cell surface‐glycans is reviewed in detail by Imberty *et* *al*.^[8]^

LecA has medium‐range affinity (K_a_ 3.4×10^4^ M^−1^) for d‐galactose (d‐Gal), however this is still tenfold higher than average affinities of C‐type lectins for their ligands and this interaction is very selective. The protein sequence and folding are unique to PA, however its binding mode is quite typical for C‐type lectins.^[20]^ LecA is highly conserved amongst clinical strains.^[12]^ High selectivity of this lectin for d‐Gal derives from H‐bonding interactions of the O2 and O3 atoms with the Asn107 residue, which itself is also involved in coordination of Ca^2+^ in the binding site (Figure 1b). O4 interacts with Thr104 and Asp100 in a similar fashion. Furthermore O6 is stabilised by interactions with a water molecule, His50 and Gln53. The shortest distance between Gal‐binding sites in LecA is ∼29 Å.

LecB is more varied in sequence and folding among clinical isolates.^[22]^ It has an unusually high affinity for fucoside terminated glycans: K_a_ with l‐fucose (l‐Fuc) is 1.6×10^6^ M^−1^, two orders of magnitude higher than LecA for Gal.^[23]^ This particularly high affinity comes at the cost of a loss in specificity; this lectin also recognises d‐mannose and d‐arabinose, among others.^[24]^ The Fuc O2, O3 and O4 atoms coordinate Ca^2+^ ions in the binding pocket and furthermore O5, O1, O2 and O3 form an intricate H‐bonding network with neighbouring residues, particularly Asp99, Asp96, Asp104, Ser23 and structural water (Figure 1d).[^8^, ^25^] The shortest distance between neighbouring Fuc‐binding sites is ∼40 Å.

While X‐ray diffraction crystallography (XRD) provides insight into ligand binding, it is not always possible to obtain this structural information, nor is it necessarily representative of ligand‐protein binding *in* *vivo*. While no single biophysical assay tells the whole story, several methods are used in conjunction to obtain complimentary information and construct a profile to characterise lectin‐inhibition.

The most commonly used assays include: Hemagglutination Inhibition Assay (HIA); Enzyme‐Linked Lectin Assay (ELLA); and competitive assays based on fluorescence polarisation (FP). HIA is a qualitative turbidimetric assay,[^26^, ^27^, ^28^] while the latter two techniques provide IC_50_ numbers as a measure of the concentration of competitive ligand added.[^29^, ^30^, ^31^, ^32^, ^33^] Isothermal Titration Calorimetry (ITC) provides dissociation constants (K_d_) of ligands to lectins as well as thermodynamic parameters for interactions in solution,[^34^, ^35^] and allows stoichiometry of interactions to be inferred. Surface Plasmon Resonance (SPR) is significantly less common, but nonetheless provides not only details on affinity but also kinetics of on‐surface interactions.[^36^, ^37^, ^38^, ^39^] ITC data in the PA lectin‐inhibitor field are particularly comparable as in most cases they were measured either by, or in collaboration with, Prof. Imberty's team at University of Grenoble‐Alps, leading to high degrees of methodological consistency across many classes of ligand described in this review. It is important to note that *precise* comparison between binding affinity values from different studies requires careful consideration, since techniques and methodologies vary. The figures in tables below are therefore provided to be illustrative of trends.

### 1.2 Lectin Inhibition as a therapeutic strategy

Lectin‐inhibition is a promising therapeutic strategy under the anti‐adhesion umbrella of infectious disease treatments. Preventing pathogen‐adhesion to host tissues and biofilm‐formation results in bacteria that are (a) unable to attack host cells; and (b) more exposed to drug treatments. Furthermore, anti‐adhesion is a non‐bactericidal strategy, which is especially attractive given the rise in antibiotic‐resistant strains in common nosocomial pathogens, including PA. Non‐bactericidal strategies do not introduce evolutionary pressure to select for resistant strains.^[40]^

There are some excellent review articles detailing advances in various treatment strategies for PA,[^5^, ^6^] as well as bacterial biofilm‐inhibition^[7]^ and antivirulence drugs and pathoblockers more generally.^[41]^ Some of these highlight people with CF specifically as an at‐risk group from PA‐infection. This is largely attributed to their expression of more Man‐ and Fuc‐terminated glycans on bronchial epithelial cells and mucins, as well as higher levels of *O*‐glycosylation, compared to the general population.[^23^, ^42^, ^43^]

LecA and LecB have been shown to inhibit Ciliary Beat Frequency (CBF) in human airway cilia *in* *vitro*,[^19^, ^44^] which makes mechanical clearance of the airway more challenging, resulting in mucus accumulation during lung infection. Treatment of affected cell cultures with l‐Fuc and d‐Gal solutions restored normal CBF.

Both lectins also play a role in lung‐injury and it was shown that monosaccharide lectin‐inhibitors reduce injury and lung bacterial load in a murine model.^[10]^ A multi‐carbohydrate solution (d‐Man, l‐Fuc and d‐Gal) also inhibits adhesion of mucoid and non‐mucoid PA strains to CF bronchial epithelial cells, and in a murine model of acute pneumonia, diminished lung‐damage, bacterial spread, and inflammatory responses. *Ex vivo* experiments on dissected murine lungs and tracheas showed it induced rapid but reversible formation of bacterial clusters, which had enhanced susceptibility to antibiotics.^[45]^

These findings are consistent with two examples of clinical application of sugar inhalation as a treatment for PA infections, namely, use of a l‐Fuc/d‐Gal solution administered by inhalation combined with Tobramycin to clear a persistent nosocomial PA infection in a 9 month old with CF;^[46]^ and a following clinical study, monitoring the effect of twice daily inhalations of the same solution on 11 adult CF patients over 21 days.^[47]^ The solution was administered as the only treatment for 4 participants and in combination with intravenous antibiotics for 7 participants. In all cases, the infection was cleared with no side effects or inflammation. Hauber and colleagues also report that, particularly when combined with antibiotics, PA counts in patients’ sputum significantly decreased, as did inflammation factor TNFα in both sputum and blood.

A recent opinion piece by Titz and co‐workers,^[48]^ inspired by entry of GMI‐1070 (a selectin antagonist) into Phase‐III clinical trials, concludes that lectin‐inhibition is a promising target for development of new anti‐microbial strategies with drug‐like properties. This is an expanding and exciting field, with potential for broader applicability, as new lectins are discovered and characterised each year, including in other pathogens.[^49^, ^50^, ^51^, ^52^]

## 2 Synthetic Monovalent and Divalent Ligands

**Figure 2:**
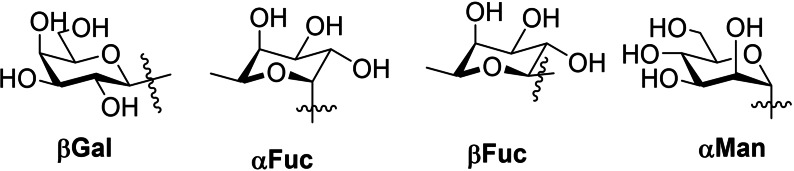
Most common monosaccharide epitopes for targeting PA lectins.

Given the promising results of studies described in Section 1.2, it is unsurprising that improvements on the affinity of the natural carbohydrates have been sought in hopes of developing new therapies and drug‐like molecules based on monosaccharides shown in Figure 2 and other natural glycans. Simple modifications, more advanced rational design, and examinations of the relationships between structure and activity have resulted in several classes of monovalent and divalent ligands which better target PA's lectins.

### 2.1 Monovalent LecA and LecB inhibitors: towards drug‐like molecules

The affinity of LecA for Gal has K_d_ of 87.5 μM. As early as 1992, specificity of this binding was investigated with a range of simple galactoside and thiogalactoside derivatives, including the widely‐used reagent **IPTG**.^[54]^ It became clear from these data that LecA had marked preference for galactoside‐derivatives featuring aromatic aglycons at the anomeric position, opening an avenue for fine‐tuning structure to create more potent ligands. *S*‐ and *O*‐aryl‐galactosides can effectively inhibit hemagglutination of erythrocytes by LecA, as well as fully inhibit its binding of labelled Gal. These compounds had K_d_ in the ∼10 μM range.^[55]^ Structures and summary of binding data are given in Table 1. In 2013, Roy developed monovalent LecA‐inhibitors with aromatic thioglycosides, seeking higher‐affinity ligands, which were stable to glycosidases.^[56]^ The best candidates identified in this study were *S*‐naphthyl galactoside and lactoside, with K_d_ of 7.9 and 5.4 μM, respectively, representing a marked but not dramatic improvement in potency.

**Table 1:** LecA affinity for selected monovalent galactosides.

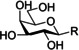					
Aglycon,^[a]^ R=	Ref.	K_d_ [μM]^[b]^	IC_50_ [μM]^[c]^	r.p.^[b]^	HIA MIC [μM]
−S^*i*^Pr (**IPTG**)	[53]	32.4		2.7	0.2
−OPh	[54]	8.8		9.9	2.1
−SPh	[54,55]	9.9		8.83	2.1
−O‐*p*‐(C_6_H_4_)‐NO_2_	[53]	14.1		6.2	0.55
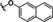	[55]	4.2		20.8	0.7
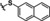	[56]	6.3	3	11.1	–
−OTol	[55]	7.4		11.8	2.1
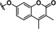	[55]	4.7		18.6	
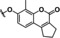	[55]	5.4		16.2	
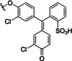	[55]	6.3		13.8	2.1
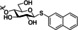	[56]	5.4	5	16.2	
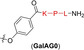	[53]	4.2		20.8	0.08
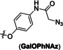	[57]	5.8	46	12.1	250
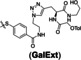	[58]	6.8		0.7

[a] Peptides are represented using one letter codes for l‐amino acids; [b] ITC; [c] ELLA.

**Figure 3:**
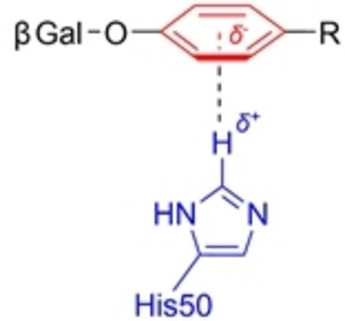
Schematic representation of “T‐shaped” CH⋅⋅⋅π interaction between aromatic aglycon and LecA's His50 residue.

Nearly simultaneously, Reymond and co‐workers delved into the nature of interactions responsible for observed affinity‐increases for Gal derivatives with aromatic aglycons, through a Structure‐Activity Relationship (SAR) study.^[55]^ A library of galactosides with *S*‐ and *O*‐ linked aglycons was analysed by ITC, and their MIC of hemagglutination determined. Additionally XRD of LecA in complex with six of these compounds revealed favourable “T‐shaped” CH⋅⋅⋅π interactions between aromatic aglycons and the proton of the His50 imidazole in the carbohydrate‐binding pocket (Figure 3) Furthermore it was observed that electron‐rich aglycons had shorter CH⋅⋅⋅π distances than their electron‐poor counterparts, which is consistent with the thermodynamic data obtained in ITC, as those with electron‐rich aglycons had stronger bonding enthalpies (around −11.5 kcal/mol). Furthermore, this study also identified five further low‐micromolar monovalent LecA ligands, including *O*‐toluyl and coumarin derivatives (Table 1).

Ligands used as monovalent “reference” compounds in order to calculate relative potencies per epitope (r.p./n) of multivalent LecA inhibitors (see Section 3) have themselves also demonstrated low‐micromolar affinities. For example, **GalAG0** the monovalent tripeptide “arm” of glycopeptide‐dendrimers (Section 3.2) has K_d_ of 4.2 μM,^[53]^ while **GalOPhNAz** used in developing glycoclusters (Section 3.4) had K_d_ of 5.8 μM and was a significantly better inhibitor than analogues with non‐aromatic flexible oligo(ethylene glycol) aglycons.^[57]^

**Figure 4:**
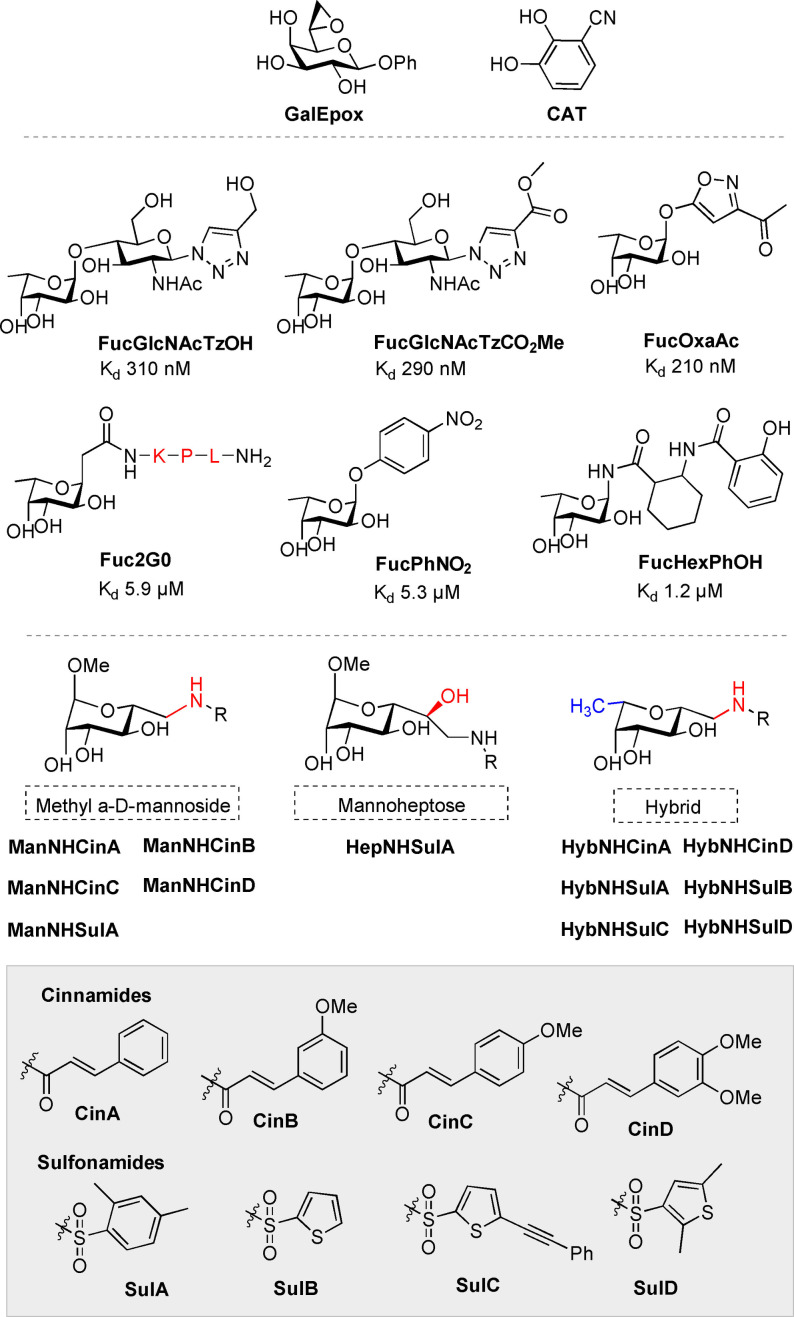
Selected monovalent inhibitors for PA lectins.

Aiming to enhance binding affinity using molecular dynamics simulation‐aided design, ligands extended to include remote aromatic groups (*e*. *g*. **GalExt**, K_d_ 6.8 μM) gave additional interactions with a central hydrophobic pocket of LecA's tetramer, confirmed by XRD and PrOF‐NMR. However no great improvement in affinity was observed because enhanced binding enthalpies were balanced by entropic penalties.^[58]^ Another approach to overcome modest affinities is installation of an electrophilic ‘warhead’ into ligand structures, to achieve persistent covalent LecA‐inhibition.^[59]^ **GalEpox** (Figure 4) was shown by mass spectrometry analysis to bind the Cys62 residue (*cf*. Figure 1b). **GalEpox** was also co‐crystallised with LecA, clearly showing proximity to Cys62. A fluorescein‐derivative was also successfully used to label the protein (see Section 4).

Finally, non‐carbohydrate catechol‐based glycomimetics were recently reported for LecA‐inhibition. XRD of **CAT**⋅LecA complex showed **CAT** bound in the carbohydrate‐binding site in an analogous way to galactosides, a first example of this type of behaviour for C‐type lectins. **CAT** had a K_d_ of 1.11 mM, (by SPR).^[60]^

No significant biofilm inhibition results are reported for monovalent galactosides. Moreover, breaking through to nanomolar affinities for LecA has proven impossible with monovalent ligands, and even development of a competitive FP assay was challenging due to the only average avidity of the interaction.^[33]^ Divalent inhibitors have proven much more successful in this regard (Section 2.2).

On the other hand, the unusually high affinity of LecB for L‐Fuc (K_d_ 2.9 μM) implies much greater potential for the development of monovalent inhibitors. In fact even a modification as simple as methylation (Me‐α‐Fuc) results in a K_d_ of 430 nM.^[24]^ The trisaccharide Lewis^a^ has been identified as the best natural ligand known for LecB (K_d_ of 210 nM); Roy developed two highly potent synthetic disaccharide ligands inspired by Lewis^a^: **FucGlcNAcTzOH** and **FucGlcNAcTzCO_2_Me** (Figure 4). Each of these ligands derives their high potency from strong interactions of Fuc along with further favourable interactions of the GlcNAc moiety's O3 group with Ser23 and O6 with Asp96 in the binding pocket.^[61]^ **FucOxaAc** was designed to mimic the GlcNAc moiety and is reported to match the affinity of Lewis^a^.^[62]^

As for **GalAG0** above, Reymond and co‐workers tested the monovalent fucosyl analogue as a LecB ligand. **Fuc2G0** had K_d_ of 5.9 μM, just slightly below that of the non‐peptidic reference **FucPhNO_2_** (K_d_ 5.3 μM) reported in the same article.^[63]^ Andreini *et* *al*. reported a family of *N*‐fucosyl amides as relatively high potency inhibitors, of which **FucHexPhOH** (K_d_ 1.2 μM) is the highest affinity ligand. The presence of the amide at the anomeric position perturbs the highly conserved H‐bonding network involving Fuc, a bound water molecule and the peptide backbone (observed in most if not all LecB‐fucoside complexes, Figure 1d) and this explains the difference between **FucHexPhOH** and higher affinity ligands.^[64]^

A downside of LecB's high affinity for αFuc is that fucoside‐glycans are ubiquitous in natural systems and can be recognised by many proteins besides the intended target. Fortunately, LecB also has a reasonably high affinity for Me‐α‐D‐mannoside (K_d_ 71 μM),^[24]^ a target that offers higher selectivity than can be achieved with αFuc, making it attractive for developing inhibitors with both high selectivity *and* high affinity, if optimised with structurally informed modifications.^[65]^

This ambitious enterprise was undertaken by Titz and co‐workers in hopes to identify drug‐like candidates. Me‐α‐D‐mannosides were modified at the primary alcohol with small libraries of triazoles, amines, amides and sulfonamides, and screened *via* FP competitive binding assays with LecB, to determine IC_50_ values (Table 2).^[32]^ While it had been anticipated that amine derivatives might form a salt bridge with Asp96, disappointing binding affinities were not supportive of this. Amine and triazole derivatives only had IC_50_ values in the 500–100 μM range. On the other hand, three amide derivatives displayed IC_50_ values <100 μM, with cinnamide **ManNHCinA** standing out at 37.4 μM (Table 2). Furthermore, all but one sulfonamide derivative were good inhibitors with IC_50_ <50 μM, *e*. *g*. **ManNHSulA**. These two high‐affinity inhibitors were identified as lead compounds and their K_d_ determined by ITC as 18.5 and 3.3 μM respectively. Both compounds feature drug‐like qualities and comply with Lipinski's rule of five, even promising oral availability according to ADMET calculations. While l‐Fuc is 26 times more potent than Me‐α‐Man as an inhibitor, **ManNHSulA** is 21.5 times more potent without being a fucoside ‐ a significant increase comparable to the natural ligand and conducive to further lead‐optimisation. XRD reveals **ManNHSulA** binds the two lectin Ca^2+^ ions through O4 and O5 in an identical fashion to Me‐α‐D‐Man. It also shows that the sulfonamide forms a H‐bond with Asp96 carboxylate in addition to lipophilic interactions of the aryl substituent with the protein surface, which help explain the excellent binding affinities obtained. Furthermore, it was demonstrated that both **ManNHSulA** and **ManNHCinA** are able to inhibit adhesion of whole PA bacteria to a fucosylated surface at 200 μM.^[32]^

**Table 2:** LecB affinity for selected monovalent ligands.

Compound	Ref.	K_d_ [μM]^[a]^	IC_50_ [μM]^[b]^	r.p./Me‐α‐Man
**ManNHCinA**	[32]	18.5	37.4	3.8
**ManNHCinB**	[66]		27.4	
**ManNHCinC**	[66]		33.6	
**ManNHCinD**	[66]	10.9	19.9	6.5
**ManNHSulA**	[32]	3.3	3.4	21.5
**HepNHSulA**	[67]		101	
**HybNHCinA**	[69]	3.09	4.21	23
**HybNHSulA**	[69]	1.27 (0.31^[c]^)	0.97 (0.34^[c]^)	55.9
**HybNHSulB**	[69]	0.83 (0.29^[c]^)	1.80 (0.44^[c]^)	85.5
**HybNHSulC**	[70]		1.52 (0.14^[c]^)	
**HybNHSulD**	[70]	1.20 (0.29^[c]^)	1.87 (0.44^[c]^)	59

[a] ITC; [b] FP assay; [c] LecB from PA14 clinical strain.

With one lead‐like compound in hand, SAR studies were undertaken to enhance the potency of mannose‐cinnamide derivatives into the low‐μM range. >20 cinnamide derivatives were made, with polar and non‐polar substituents at *ortho‐*, *para‐* and *meta‐*positions, doubly‐substituted derivatives and a 2‐naphthamide, to probe the role of the aromatic moiety in LecB interactions.^[66]^ In general, of the singly‐substituted cinnamides, it was observed that *ortho*‐substituted derivatives were weaker inhibitors compared to *meta*‐ or *para*‐regioisomers, perhaps owing to steric hindrance within the binding pocket. Any polar substituents resulted in weaker inhibitors than lipophilic substituents, but particularly in *meta‐* and *para‐* positions. The best substituent identified was methoxy (**ManNHCinB‐D**). Di‐methoxy‐derivative **ManNHCinD**, was the most potent inhibitor of the study with IC_50_ of 19.9 μM, enhanced compared to singly substituted methoxy‐derivatives (Table 2). XRD analysis of a **ManNHCinA**⋅LecB complex revealed that the binding mode for this interaction was different to that observed for **ManNHSulA** and affinity stemmed largely from hydrophobic interactions between Gly97, Thr98 and the cinnamide moiety as well as H‐bonding between the amide and Ser23. Investigation into kinetics of interactions between LecB and **ManNHCinD**, **ManNHSulA** or **ManNHCinA** revealed that ligand‐protein interactions were long‐lived. The half‐lives were much longer than the 0.75 min reported for Me‐α‐D‐Man, particularly in the case of **ManNHSulA** which displayed a half‐life of 18.64 min, a 24‐fold improvement. The long‐lasting nature of this interaction is a further indicator of drug‐like properties.

A family of mannoheptoses with cinnamide and sulfonamide substituents was also synthesised by extending the sugar in hopes of increasing affinity by freeing the O6 position to form H‐bonds with Ser23, as seen interactions with natural d‐Man. However, these ligands, such as **HepNHSulA**, do not represent an improvement in affinity over their mannose‐analogues and this strategy was less impactful than anticipated.^[67]^

Although low‐micromolar affinities were achieved with Man‐based inhibitors, the carbohydrate‐recognition domain of LecB is highly specific for fucosides. At a point where no further optimisation of Man‐based compounds could be achieved, a biophysical study comparing the binding properties of various Fuc and Man derivatives identified the molecular origins of this selectivity and the most favourable interactions in the binding pocket, evaluating individual contributions of each substituent group to the binding. From this panel of derivatives, a hybrid bespoke glycomimetic featuring the CH_3_ group present in Fuc and the O6 terminus of Man was identified as “the best of both worlds”, allowing for the lipophilic interaction of the methyl group, as well as the O6‐Ser23 H‐bonding interaction observed for Man. This hybrid‐glycomimetic is conducive to further drug design as substituents can be introduced that result in interactions with the nearby Asp96 and hydrophobic interactions, in addition to the already enhanced affinity, as discussed for sulfonamide‐derivatives of Man above.^[68]^

Sulfonamide‐ and cinnamide‐derivatives of the hybrid‐glycomimetic such as **HybNHCinA**, **HybNHSulA**, and **HybNHSulB**, (all analogues of previously identified Man‐based lead compounds) showed improved binding affinities and anti‐biofilm activity against both the common reference strain PAO1 and the more virulent PA14 strain (reduction of biofilm by >75 % with respect to the control). All were, in fact, more potent inhibitors of LecB from PA14, reaching <450 nM affinities in the case of both sulfonamide derivatives by two separate assays; their affinities for the wild‐type LecB (PAO1) are also excellent (Table 2). Inhibitors showed no toxicity and good selectivity for LecB even in the presence of langerin (a human C‐type lectin selective for Fuc and Man). *In vitro* ADME experiments showed good metabolic stability of the inhibitors against liver microsomes and murine and human plasma, as well as promising oral availability in a murine model. High concentrations measured in the plasma of the mice and a urinary excretion pathway presents the possibility to use these compounds against PA urinary‐tract infections. Furthermore, SPR measurements revealed a half‐life of 28 and 28.2 min respectively for **HybNHCinA** and **HybNHSulA**. Another improvement with respect to their previously discussed Man‐analogues and confirmation of their drug‐like properties.^[69]^

A hydrophobic pocket identified close to the binding site offers opportunities for additional lipophilic interactions to further improve the potency of these highly selective compounds.^[70]^ Extending the hydrophobic thiophene moiety, *e*. *g*. **HybNHSulC**, leads to higher affinity than **HybNHSulB** against LecB from PAO1, but the impact on the PA14 strain is more dramatic (tenfold), with nanomolar affinities achieved (Table 2). **HybNHSulC** with a PA14 IC_50_ of 140 nM is the best in a library of 44 screened candidates, most of which show the same trend between the two strains. Furthermore, **HybNHSulC** and **HybNHSulD** showed excellent biofilm‐inhibition properties (∼85 and ∼75 % inhibition respectively, relative to control) and stability in mouse blood plasma over 2 hours with very low to no toxicity in both human and murine liver cells even at concentrations as high as 100 μM. Exploiting knowledge of interactions in the binding‐pocket of LecB to inform design of new LecB‐selective inhibitors in a drug‐like approach is proving to be an excellent way to obtain high potency monovalent ligands.

### 2.2 Divalent inhibitors: role of linker length and rigidity

**Figure 5:**
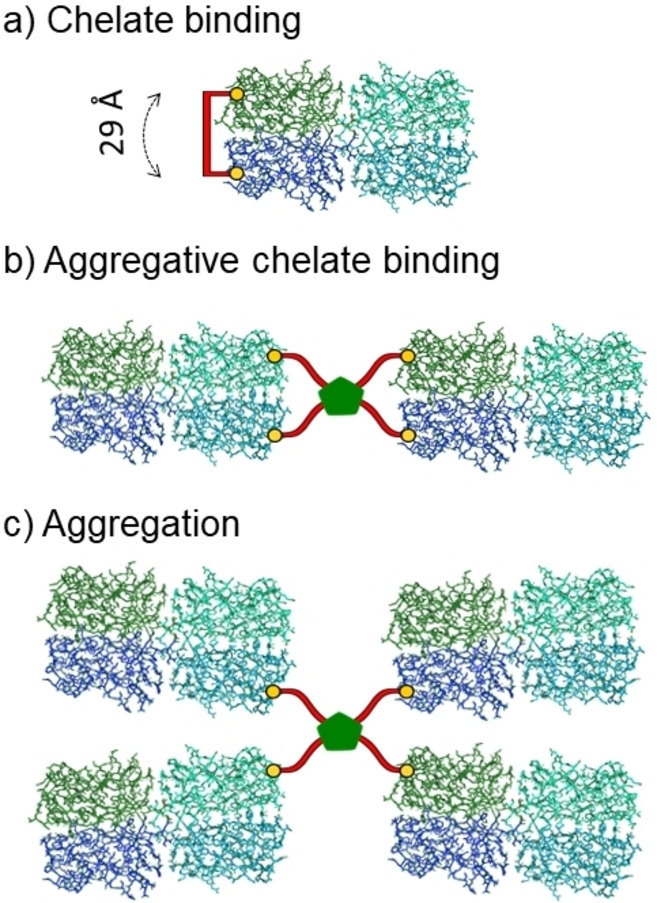
Illustration of some potential binding modes for galactosides with LecA: **a)** chelate binding mode with divalent inhibitor; **b**–**c)** aggregative chelate and aggregation with tetravalent inhibitors. (adapted from ^[75]^)

While many excellent monovalent inhibitors for LecB have been developed, similar work with LecA is not prevalent for reasons explored above. Introducing multivalency into inhibitor‐design can boost the strength of interactions. Pieters and co‐workers, in particular, have carried out significant work understanding and optimising the role of linker‐length, rigidity and multivalency on increasing LecA binding affinity of glycoconjugates. Bridging adjacent carbohydrate‐binding sites has proven an effective path to more potent inhibitors, capable of chelating the two nearest binding sites, Figure 5a.^[71]^ These sites are *ca*. 29 Å apart, as measured from XRD (*cf*. Figure 1a). Microarrays of different valency glycodendrimers were tested with LecA and observed multivalency effects correlated well with this inter‐binding site distance; di‐ and tetravalent compounds showed strong interactions with the lectin. ELLA assays showed low‐micromolar affinity (16‐fold enhancement per d‐Gal).^[72]^

Pieters argued that the role well‐defined spacers has been underestimated in LecA‐inhibitor design and that candidates with flexible polyethylene glycol‐based spacers, such as **DivalPEG**, may not be appropriate for effective binding, giving only millimolar affinities, despite having appropriate length to chelate binding sites (Table 3).^[73]^ As part of a rational design strategy, a rigid spacer with alternating 1,2,3‐triazole (Tz) and 1–4‐linked glucose components was described, with overall linear geometry and good water solubility.^[74]^

**Table 3:** LecA affinity for selected divalent galactosides.

Compound	Ref.	Scaffold	K_d_ [nM]	IC_50_ [nM]	r.p./n
**DivalPEG**	[73,74]	PEG	1900^[a]^	2000^[b]^	30^[b]^
**U2C1**	[74]	Glc‐triazole		3500^[b]^	17^[b]^
**U3C1**	[74]	Glc‐triazole	28^[a]^	2.7^[b]^	111^[a]^ 3778^[b]^
**U3C3**	[74]	Glc‐triazole	130^[a]^	120^[b]^	968^[b]^
**U3C1‐pyr**	[76]	Glc‐triazole	89^[a]^	5.2^[b]^	
**U3C1‐NH_2_**	[76]	Glc‐triazole	56^[a]^	19^[b]^	
**U2PhGal**	[78]	Glc‐triazole	35^[a]^		120^[a]^
**U3Th**	[79]	Glc‐thiourea	30^[a]^		
**U3Ph**	[78]	Glc‐triazole‐Ph	12^[a]^		258^[a]^
**U3Bu**	[78]	Glc‐triazole‐Bu	13^[a]^		238^[a]^
**Bis(U3Ph’)**	[80]	Glc‐trizole+PEG	59^[a]^		53^[a]^
**TznA**	[81]	Trithiotriazine	1090^[a]^		29^[a]^
**TznB**	[81]	Trithiotriazine	3400^[a]^		14^[a]^
**PEPb**	[83]	Peptide	82^[a]^		43^[a]^
**B5*p***	[84]	Benzylhydrazone	10.8^[c]^		259^[c]^
**C5*p***	[84]	Benzylhydrazone	20.5^[c]^		137^[c]^
**B5*m***	[84]	Benzylhydrazone	27.3^[c]^		90^[c]^
**C5*m***	[84]	Benzylhydrazone	18.9^[c]^		130^[c]^

[a] ITC; [b] Galactose‐functionalised surface ELISA type assay^[73]^; [c] SPR.

**Figure 6:**
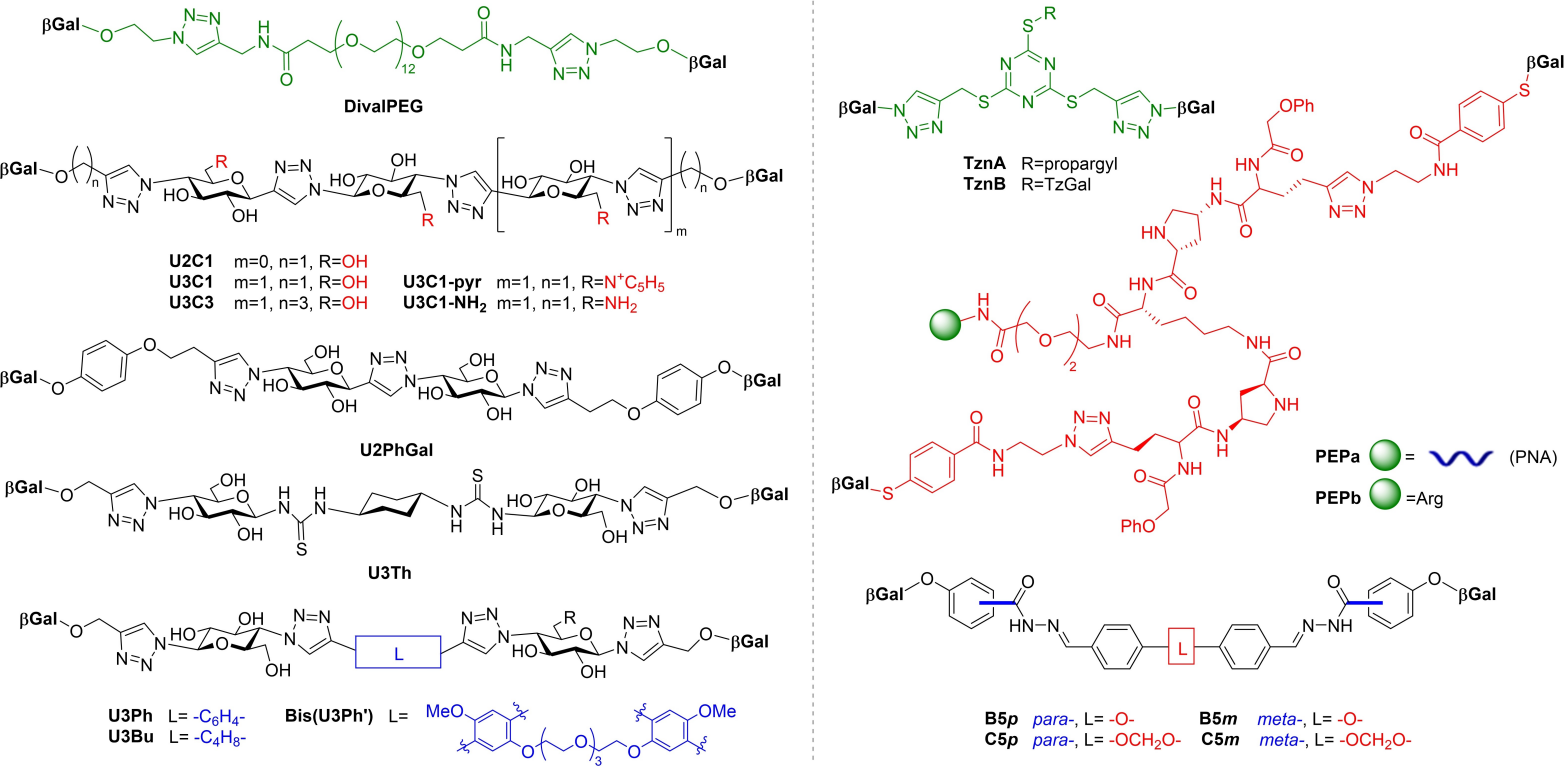
Selected divalent galactoside LecA‐inhibitors, with various degrees of rigidity.

Compounds with three spacer‐units proved most effective, with nanomolar affinities (Table 3) determined by both an ELISA‐type assay and ITC, while increasing or decreasing spacer‐length pushed affinities back into the millimolar range. Upon adding a triazole‐glucose unit to the structure, going from **U2C1** to **U3C1**, a relative potency enhancement per galactose from 17 to 3778 was obtained. ITC studies gave a stoichiometry of ∼0.5, consistent with divalent chelate lectin‐binding (Figure 5a), and the difference between the two best inhibitors, **U3C1** and **U3C3** (varying only in aglycon length), were almost exclusively entropic; **U3C1** (K_d_ 28 nM) benefitted from its enhanced rigidity (Figure 6). This shows how delicate the balance is for optimal tuning of the spacer for such rigid systems, but also the dramatic and beneficial impact structural tuning can have.^[74]^

Molecular modelling suggested that the C6 position of flanking Glc‐units was appropriately positioned to interact with Asp47 in the LecA structure (*via* its carboxylate moiety). Experimental binding data of C6‐modified structures, however showed minimal impact of positively‐charged groups at this position (**U3C1‐pyr** and **U3C1‐NH_2_** in Table 3), suggesting that such protein‐ligand interactions are of minimal importance in solution.^[76]^

XRD of **U3C1**⋅LecA complex confirms the chelate‐binding mode proposed by molecular modelling and ITC.^[77]^ Interestingly, unlike other aromatic aglycons (see Figure 4) the triazole does not form CH⋅⋅⋅π interactions with His50.^[55]^ This is perhaps due to favourable H‐bonding interactions between flanking glucose units and structural water molecules on the protein surface, a bonding network including His50, Tyr36 and Gln40. While replacing triazolyl aglycons with phenyl does indeed enhance enthalpy of binding for rigid divalent inhibitors of similar length, such as **U2PhGal**, concomitant entropic losses mean that this series of inhibitors is less potent. Entropic penalties of protein rearrangement negate any advantage of this common strategy for K_d_ enhancement.^[55]^ Solubility was also an issue for some phenylene‐derivatives.^[78]^ In this regard, these rigid structures are an atypical example of a high‐potency LecA‐inhibitor, which benefits from structural features other than aromatic aglycons.

An analogue of **U3C1**, replacing central bis(triazolyl)glucose motif of with a bis(thiourea)cyclohexyl group, yielded **U3TU** with equivalent LecA binding potency, and with significantly simplified core synthesis (halving the number of steps to 7).^[79]^ Replacing this core with a phenyl ring, **U3Ph**, led to increased affinity, with increased r.p./n of 258 (compared to 111 for **U3C1**), which remains among the highest enhancements reported, even including multivalent systems (Section 3 below).^[78]^ More flexible butyl‐centred linkers (**U3Bu**) gave similarly impressive results.

**U3C1** and **U3C3** showed weak biofilm‐inhibition (50 % at 150 μM), comparing very poorly with tetravalent glycoclusters **GalAG2** (*vide infra*). This points to the importance that cross‐linking lectins typically has, in addition to chelate‐binding, when it comes to anti‐biofilm therapeutic activity.^[77]^ Indeed, a tetravalent derivative, **Bis(U3Ph’)** could much more effectively disperse or inhibit biofilms, giving 46 % inhibition at only 28 μM.^[80]^ Stoichiometry of interaction determined from ITC supports 1 : 4 interactions, aggregating two LecA tetramers, as illustrated in Figure 5b. This study gratifyingly shows the power of rational inhibitor‐design, informed by lectin structure and binding‐site topology.

Low‐valency galactoclusters **TznA‐B** based on dendritic trithiotriazine cores with inter‐galactoside distances less than 29 Å, unsurprisingly had modest affinities. Fucoside analogues also showed poor LecB binding, however these clusters could inhibit PAO1 biofilm formation at 5 mM.^[81]^ Another class of divalent fucosides with flexible linkers were shown to have good affinity for LecB (up to 90 nM), but due to the longer distance between LecB binding sites chelate‐binding was not achieved, only cross‐linking of neighbouring tetramers.^[82]^

A galactoside‐conjugate DNA‐based array was used to identify a potent LecA inhibitor from a library of 625 divalent candidates, with ligand design informed by the structural considerations already discussed. **PEPa** was identified by this method, and ITC of its arginine‐derivative **PEPb** showed a K_d_ of 82 nM. **PEPa** (when hybridized with its complementary DNA) showed the ability to block cellular invasion by PA in human lung epithelial cells by 80–90 % at as low as 50 nM (compared to modest protection at 10 mM by monovalent galactoside).^[83]^

Recently, Titz and co‐workers reported a remarkably elegant route to low nanomolar divalent LecA‐inhibitors, requiring only four synthetic steps from pentaacetylated d‐Gal. Using phenylgalactoside as a LecA targeting motif, *para*‐ and *meta‐*hydrazides were reacted with bis‐benzaldehydes of varying spacer‐length. All examples had nanomolar affinity, with linker‐length playing a key role in fine‐tuning K_d_. The shortest example in the *para‐*family, **B5*p*** proved the most potent divalent LecA ligand to date (K_d_ 10.8 nM, Table 3); molecular modelling indicated that spacers were of appropriate length to bridge LecA's adjacent galactose‐binding sites. Selectivity of this ligand for LecA over human galectin‐1 was also established. The synthetic simplicity of this design is very promising for future optimization of structures, even potentially to enhance their drug‐like properties.^[84]^

## 3 Approaches to Multivalent Glycocluster Design

**Figure 7:**
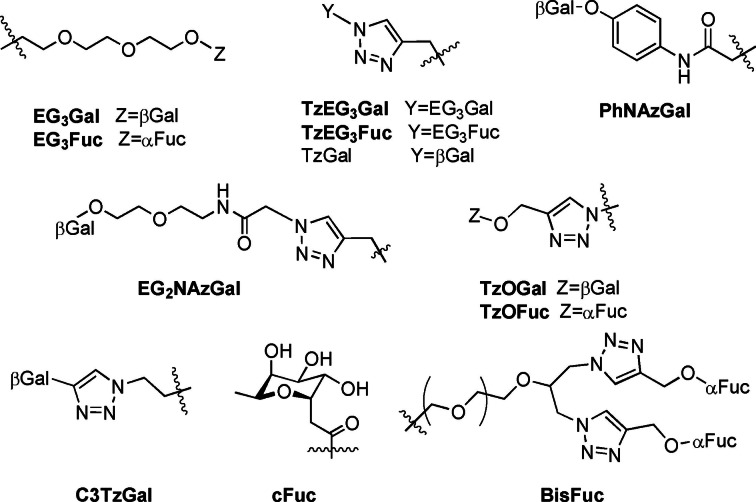
Common linkers in PA lectin‐inhibitor design.

Multivalency effects are a reliable way to increase avidity of systems for carbohydrate‐binding proteins,^[85]^ although it has been pointed out that such large molecules may present pharmaceutical challenges in delivery or side‐effects.^[86]^ The vast majority of glycoconjugates reported as LecA‐ and LecB‐inhibitors are multivalent glycoclusters, based on widely varying libraries of scaffolds in order to control presentation of carbohydrate epitopes. The following sections will highlight major glycocluster families, and compare advantages and disadvantages. For convenience, several common linker groups utilised in multivalent systems are defined in Figure 7.

### 3.1 Multivalent systems immobilised on nucleotide support

**Figure 8:**
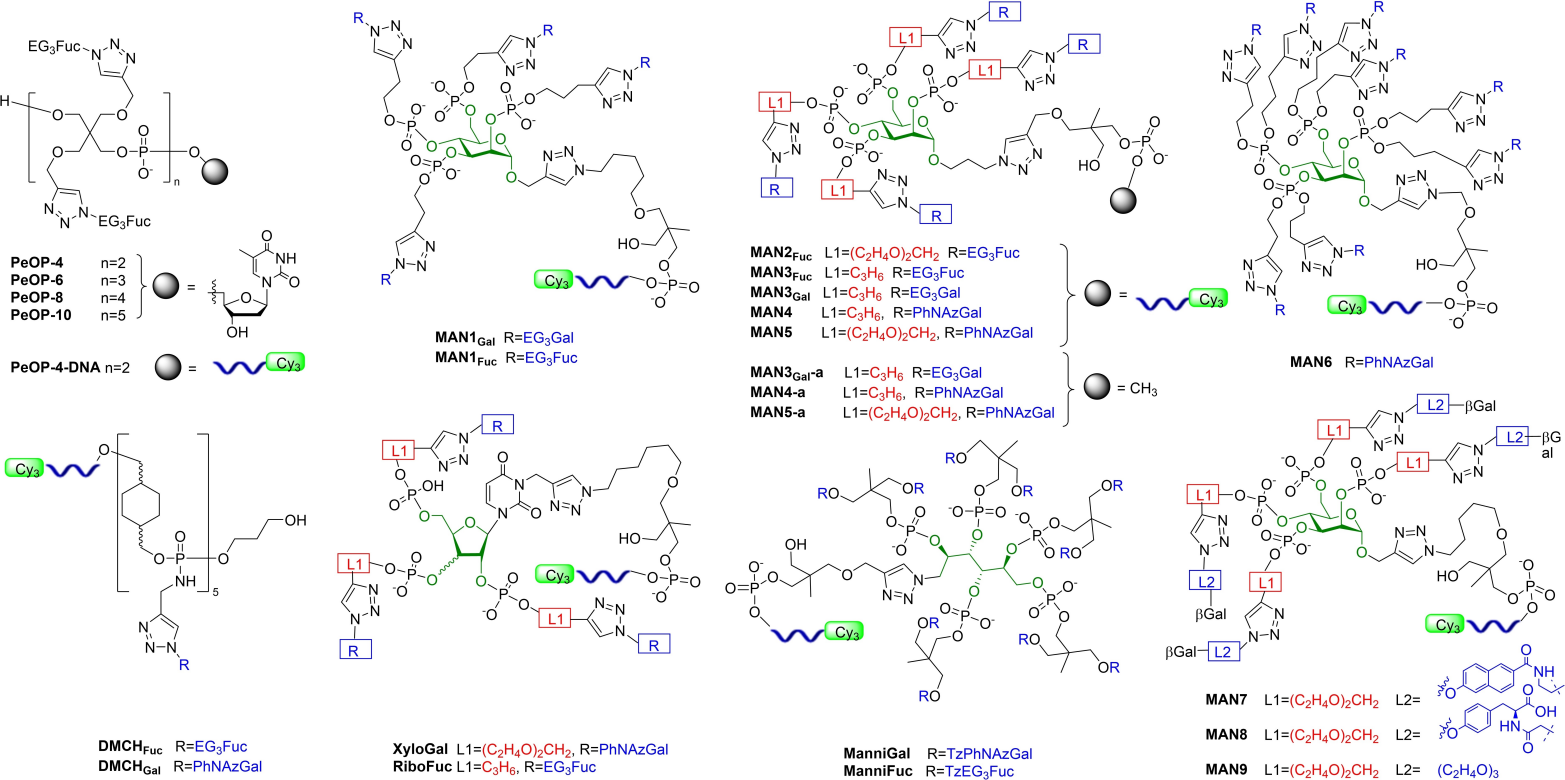
Selection of inhibitors inspired by DNA‐synthesiser reactions.

Reactions and linkages widely employed in DNA‐synthesiser technology were adopted as a source of inspiration for modular inhibitor design by Morvan, Vidal and co‐workers. In their first article on this topic, they construct glycoclusters from building‐blocks, consisting usually of glycosides (with linker), phosphoramidite tethers, a scaffold, and nucleotides or a short strand of synthetic DNA (Figure 8).^[87]^ Components are assembled through well‐known and reliable reactions; phosphoramidite chemistry (using a DNA‐synthesiser) for the pentaerythriyl‐phosphodiester core, and microwave‐assisted CuAAC to conjugate glycosides giving clusters such as **PeOP** (4‐, 6‐, 8‐ and 10‐valent fucoclusters).^[87]^ In a binding competition assay vs L‐Fuc, all exhibit sub‐micromolar IC_50_ for LecB, Table 4. There is a clear relationship between valency and inhibition, however the increase in r.p./n is not particularly impressive, and not strong enough to be attributed to a “cluster” effect. The best compound of this family was decavalent **PeOP‐10** (IC_50_ 250 nM).

**Table 4:** Affinity of selected phosphodiester‐containing clusters.

Compound	Ref.	Scaffold	Lectin	K_d_ [nM]	IC_50_ [μM]	r.p./n
**PeOP‐10**	[87]	Pe	LecB		0.25^[a]^	2.2
**PeOP‐4‐DNA**	[90]	Pe	LecB		15^[b]^	18.8^[b]^
**MAN2**	[90]	Man	LecB		14^[b]^	17.5^[b]^
**MAN3_Fuc_**	[90]	Man	LecB		11^[b]^	13.8^[b]^
**DMCH_Fuc_**	[90]	DMCH	LecB		8^[b]^	8^[b]^
**DMCH_Gal_**	[91]	DMCH	LecA		1550^[b]^	62^[b]^
**MAN3_Gal_**	[91,92]	Man	LecA	395^[c]^	29^[b]^	1.5
**MAN4**	[91,92]	Man	LecA	60^[c]^	2826^[b]^	141
**MAN5**	[91,92]	Man	LecA	39^[c]^	4218^[b]^	211
**MAN6**	[91]	Man	LecA		6803^[b]^	170^[b]^
**MAN3_Gal_‐a**	[92]	Man	LecA	11000^[d]^	27.6^[a]^	1.6
**MAN4‐a**	[92]	Man	LecA	194^[d]^	0.26^[a]^	90^[d]^
**MAN5‐a**	[92]	Man	LecA	157^[d]^	0.06^[a]^	111.5^[d]^
**XyloGal**	[93]	Xylo	LecA	49^[c]^		
**ManniGal**	[93]	Mannitol	LecA	50^[c]^		
**ManniFuc**	[93]	Mannitol	LecB	84^[c]^		
**RiboFuc**	[93]	Ribo	LecB	56^[c]^		
**MAN7**	[94]	Man	LecA	20^[c]^	180000^[b]^	
**MAN8**	[95]	Man	LecA	19^[c]^		
**MAN9**	[13]	Man	LecA	395^[c]^

[a] ELLA; [b] Competitive on‐surface assay, where higher values indicate stronger binding;^[90]^ [c] K_d_ determined on array;^[95]^ [d] ITC.

Following from this, DNA‐Directed Immobilisation (DDI) technology was adopted to rapidly assess libraries of surface‐bound glycoclusters as ligands for PA lectins *via* glycan microarray approach. An in‐depth discussion of DDI technology, design and applications can be found in Morvan's review.^[88]^

Mannose‐centred glycoclusters were the first instance of carbohydrates as scaffolds for PA lectin‐targeting. Tethered *via* DDI at the anomeric position, the four remaining hydroxy‐groups were functionalised with EG_3_‐linked monosaccharides to yield tetravalent clusters **MAN1** with a defined topology provided by the mannose core. A heterocluster, combining both **MAN1_Gal_** and **MAN1_Fuc_** through a long flexible linker, was also synthesised. All three ligands were screened for activity with LecA and LecB through a fluorescence assay, with heterocluster having a marked increase in fluorescence in the presence of both lectins (rather than only one), and much higher than the homoclusters in all instances. However, no quantitative binding measurements were reported.^[89]^

A series of sixteen fucosylated ligands was synthesised, varying the topology and spatial arrangement.^[90]^ This small library featured monovalent compounds; linear compounds of various valencies based on dimethanolcyclohexane (DMCH), and bis‐pentaerythrityl (Pe) scaffolds; and finally carbohydrate‐scaffolds based on Man, Gal and d‐glucose (Glc). In addition to presentation topology, the influence of linker‐length was also studied, with 13, 17 and 21 atoms between the alcohol groups of the hexose scaffold and the anomeric oxygen of the Fuc epitope. An on‐surface assay was developed by the group in this work: the clusters immobilised on glass slides and tagged with Cy3 were incubated with Alexa 647‐tagged LecB, then increasing concentrations of Fuc were added to determine IC_50_ by measuring the decrease of the Alexa 647 fluorescence signal. Therefore, in this case higher IC_50_ values indicate *better* binding of the cluster to target lectin, as a higher concentration of the natural ligand was needed to displace 50 % of the interactions. Selected IC_50_ values are shown in Table 4. Tetravalent compounds **PeOP‐4‐DNA** and **MAN2**, based on two different scaffolds, were the standouts from the study (IC_50_ 15 and 14 μM, respectively). It was observed that among the carbohydrate‐centred fucomimetics, those based on Man cores, such as **MAN3_Fuc_** performed significantly better than analogues based on Gal or Glc scaffolds (IC_50_ of 11, 1 and 2 μM, respectively), indicating benefits of Man‐topology, however the **PeOP‐4‐DNA** resulted in the greatest enhancement in binding (r.p./n of 18.8). It was also pointed out that longer linkers seem to positively influence binding, as among the Man‐centred compounds r.p. increases with linker length and across the board compounds with a 21‐atom linkers (*e. g*. **MAN2**) performed better than those with shorter linkers (*e. g*. **MAN3_Fuc_**). Despite good activity and a multivalent presentation, the influence of multivalency seems to be less pronounced, as expected, in the case of LecB, as no chelate‐binding was observed.

SAR of a library of 25 DDI‐immobilised galactomimetics, based on linear and hexose‐centred scaffolds allowed the influence of linker‐length and topology on LecA‐binding to be explored. The modular components of this library also allowed the role aromatic galactoside‐aglycons to be assessed efficiently.^[91]^ An on‐surface competitive fluorescence assay^[90]^ was used to obtain IC_50_ values, using lactose as competitive inhibitor (Table 4). **MAN3_Gal_**, with EG_3_‐aglycon gave relatively poor binding. By contrast, four compounds, with phenyl aglycons excelled. Two tetravalent Man‐centred clusters, **MAN4** and **MAN5**, where *L1*=propylene or EG_2_, respectively (Figure 8), and octavalent **MAN6**, all showed high affinity for LecA and nanomolar K_d_ determined on the microarray.^[92]^ A pentavalent linear cluster based on repeating DMCH units, **DMCH_Gal_**, was also an effective LecA‐inhibitor. These four standout compounds all feature *O*‐phenyl aglycons. As discussed in Section 2.1, it was clear from the data that aromatic aglycons increase affinity considerably,^[55]^ and phenyl aglycon outperforms Tz analogues.[^8^, ^80^, ^90^]

Non‐immobilised analogues of the highest‐affinity tetravalent systems **MAN4‐a** and **MAN5‐a** showed similar affinity trends in solution, and were demonstrated to inhibit PAO1 biofilm formation by 40 %, at 10 and 5 μM, respectively. These compounds could also disperse established biofilms.^[92]^

Man‐centred clusters had higher affinity for LecA in general (compared to Gal or Glc), indicating that scaffold‐topology is important, however an interesting observation was made on the relationship between the nature of the linker and the scaffold: with shorter linkers Man‐clusters were better, however with longer more aliphatic linkers Glc‐clusters had higher affinities. This indicated that linker‐length and presentation‐topology, act together rather than separately to influence affinity.^[91]^ In general, however, longer more hydrophilic linkers at *L1*, such as oligoethyleneglycols, give better results. Higher valency is also beneficial in this case, as demonstrated by the 2.4‐fold increase in affinity of **MAN4** vs **MAN6**, respectively tetra‐ and octavalent clusters with the same combination of features.

The importance of carbohydrate‐scaffold topology was further explored by synthesis of two further families of clusters: trivalent furanose‐based systems (arabinose, ribose and xylose); and open chain penta‐ and decavalent mannitol systems.^[93]^ A total of 9 galacto‐ and 9 fuco‐clusters, were synthesised modularly, and K_d_ determined. In this study, all glycoclusters screened exhibited nanomolar affinities, and activity differences were subtle. For LecA‐targeting clusters, xylose‐based cores were generally preferred, and longer hydrophilic linkers at *L1* were slightly more advantageous (Figure 8). Benefits of specific topologies over “cluster” effects are also much more evident as the decavalent mannitol cluster **ManniGal** and the best trivalent cluster **XyloGal** had almost equivalent K_d_ (Table 4). For LecB‐targeting clusters, ribose and xylose‐based cores exhibited very similar behaviours, but arabinose was markedly disfavoured. Linkers had little to no effect and multivalency was proven irrelevant by the fact that the decavalent cluster **ManniFuc** was a worse inhibitor than any furanose‐based cluster. Among fucomimetics, **RiboFuc** was best, illustrating well the more pronounced importance of multivalency effects when targeting LecA vs LecB.

Enhancement of affinity for LecA upon incorporation of either *S*‐ or *O*‐linked aromatic aglycons was again seen in a study of linker impact on Man‐centred clusters (*L1* and *L2*, Figure 8).^[94]^ These compounds showed nanomolar K_d_, but ligands with shorter EG_2_‐linkers at *L1* alongside naphthalene and diphenyl derivatives (*e. g*. **MAN7**) showed highest affinity (K_d_ 20 nM). This structural combination maintains the total length between triazole and the epitope's anomeric oxygen close to the optimum of *ca*. 25 atoms, the same length as was achieved with phenyl‐aglycons at *L2* and a longer linker at *L1* (*e. g*. **MAN4**). Linking carbohydrates to mannose scaffolds through phosphodiester rather than phosphorothioate‐linkages seems to also have beneficial effects on K_d_. Overall it was determined that, of the four aglycons used, the order of influence on affinity is benzyl<phenyl<biphenyl≤naphthyl. Further structural optimization in this vein screened 27 compounds, with a wider range of linkers (*L1*) and aglycons (*L2*), concluding that EG_3_ is the best *L1*, as it offers hydrophilicity, flexibility and optimum length in most combinations.^[95]^ Nine different aglycons at *L2* explored influence of ring‐size, heteroatoms, distance between ring and epitope, and regio‐isomerism of substitution. The influence of *L2* on binding affinity was greater than *L1*, because *L2* can interact with the lectin binding‐site. **MAN8**, with tyrosine‐derived aglycon possessing a free carboxylate was a low‐nanomolar LecA ligand, outperforming the regioisomeric compound with a free amine group, likely due to carboxylate interaction with His50 rather than π‐stacking (as observed for phenyl aglycons). Docking simulations of **MAN8** with LecA reveal two additional possible stabilising interactions with Pro38 and Glu39.

Multiple functionalisations of phosphodiester moieties were only implemented in octavalent compound **MAN6**, where Angeli *et al* explored whether adding additional chains would affect binding through complementary lectin‐interactions in the binding‐site. Hydrophobic and hydrophilic chains with amide and amine functionalities were utilised. It was challenging to achieve significant affinity increases in this way when aromatic aglycons were present, but in structures with a non‐aromatic aglycon (i. e. EG_3_ at *L2*), the impact of a secondary chain could improve affinity to the levels of clusters with aromatic aglycons. For instance, the K_d_ of cluster **MAN9** was enhanced from 395 nM to 48 nM upon introduction of a (C_2_H_4_O)_2_C_2_H_4_NH_3_^+^ chain, indicating that this alternative strategy for mannose‐centred clusters also has merit.^[13]^

### 3.2 Peptide and β‐peptoid‐based scaffolds

**Figure 9:**
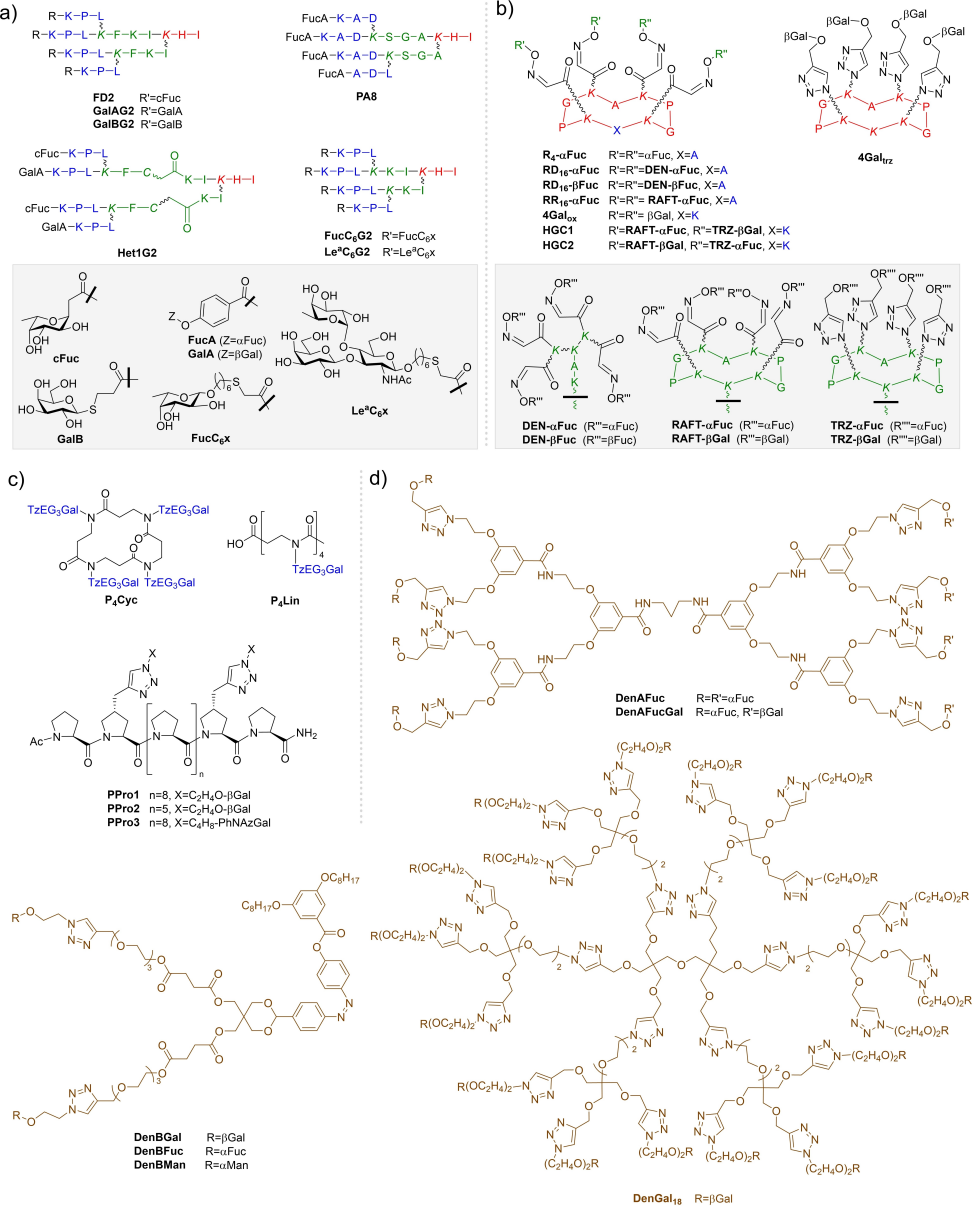
Selected dendrimer structures: **a**–**b)** Glycopeptide dendrimers represented using one letter codes for l‐amino acids, wobbly bonds mark side‐chain lysine connectivity, and lysine (*K*) indicated in italics are branching‐points. Various generations of dendrimer synthesis are indicated in different colours for clarity. **c)** β‐peptoid glycoclusters and polyproline helices; **d)** Non‐peptide dendrimers.

A significant number of PA lectin‐inhibitors based on glycopeptide dendrimer scaffolds are reported, with lysine residues acting as branching points (Figure 9a). This approach (up to 2013) was thoroughly reviewed by Reymond and co‐workers.^[96]^ In the seminal article, a library of >15,000 fucosylated glycopeptide dendrimers (up to 4 generations) were rapidly synthesized *via* solid‐phase peptide synthesis (SPPS), followed by screening on solid‐support beads with Rhodamine B‐labeled lectins.^[97]^ Lead candidates from this library were re‐synthesised, cleaved from the solid support to determine lectin‐affinity and anti‐biofilm activity; data for some of the best‐performing examples are given in Table 5. Tetravalent 2^nd^‐generation dendrimer **FD2** (Figure 9a) was determined to have affinity for LecB almost 80‐fold that of Fuc. **FD2** also showed very potent activity for biofilm‐inhibition and dispersion of established biofilms with both PAO1 and also antibiotic‐resistant clinical isolates.[^63^, ^97^, ^98^, ^99^] In knockout strains, not containing the *lecB* gene, **FD2** showed no effect, supporting the hypothesis that activity is LecB‐mediated.^[96]^ The analogue of **FD2** with unnatural d‐amino acids (**d‐FD2** ^[100]^) was more resistant to proteolytic degradation than **FD2**, but its LecB affinity was 5‐fold weaker and it was less active against clinical isolates. A second library, bearing hydrophobic aromatic residues next to Fuc was also studied (*e. g*. **PA8**), but this modification was not found to significantly enhance affinity for LecB, in contrast to observations for other lectins.^[63]^

**Table 5:** Affinity and biological activity of selected glycopeptide dendrimers.

Compound	Ref.	Scaffold	Lectin	K_d_ [nM]	IC_50_ [nM]	r.p./*n*	Activity against PA		
							MBIC [μM]	MBC (μM)	Biofilm Dispersal^[c]^
**FD2**	[63,97,102]	Peptide dendrimer	LecB	66^[a]^	140^[b]^	19.7^[b]^ 2.9^a^	20	>30	100 %
**PA8**	[63]	Peptide dendrimer	LecB		110^[b]^	25^[b]^	50		35 %
**d‐FD2**	[100]	Peptide dendrimer	LecB		660^[b]^	4.2^[b]^			80 %
**2G3**	[98]	Peptide dendrimer	LecB		25^[b]^	55^[b]^			
**GalAG2**	[53,102]	Peptide dendrimer	LecA	100^[a]^		219^[a]^	20	>30	100 %
**GalBG2**	[53,102]	Peptide dendrimer	LecA	400^[a]^		60^[a]^	20		60 %
**GalA‐KPL**	[101]	Monovalent tripeptide	LecA	4300^[a]^		20^[a]^			
**GalA‐KPY**	[101]	Monovalent tripeptide	LecA	2700^[a]^		33^[a]^			
**GalA‐KRL**	[101]	Monovalent tripeptide	LecA	2700^[a]^		33^[a]^			
**G2KPY**	[101]	Peptide dendrimer	LecA			1700^[d]^	30		80 %
**G2KPW**	[101]	Peptide dendrimer	LecA			830^[d]^	20		70 %
**Het4G2**	[102]	Peptide dendrimer	LecA	120^[a]^		153^[a]^	>45	>45	0 %
			LecB	121^[a]^		3.2^[a]^			
**Het2G2**	[102]	Peptide dendrimer	LecA	75^[a]^		20^[a]^	30	>45	35 %
			LecB	292^[a]^		1.93^[a]^			
**FucC_6_G2**	[102]	Peptide dendrimer	LecB	121^[a]^		0.9^[a]^	9	>20	100 % (at 30 μM)
**Le^a^C_6_G2**	[102]	Peptide dendrimer	LecB	39^[a]^		2.8^[a]^	30		88 %
**GalAxG3**	[103]	Peptide dendrimer	LecA	2.5^[a]^		148^[a]^	9		100 %
									
**R_4_αFuc**	[104]	Cyclopeptide	LecB		145^[b]^	1^[b]^			
**RD_16_αFuc**	[104]	Cyclopeptide+peptide dendrimer	LecB	28^[a]^	0.6^[b]^	64.6			
**RR_26_αFuc**	[104]	Cyclopeptide	LecB		7.6^[b]^	5^[b]^			
**RD_16_βFuc**	[104]	Cyclopeptide+peptide dendrimer	LecB	213^[a]^	109^[b]^	40			
**4Gal_ox_**	[105]	Cyclopeptide	LecA	91^[a]^		412^[a]^			
**4Gal_trz_**	[105]	Cyclopeptide	LecA	22^[a]^		1705^[a]^			
**HGC1**	[105]	Cyclopeptide	LecA	34^[a]^		0.8^[a]^			
			LecB	92^[a]^		551^[a]^			
**HGC2**	[105]	Cyclopeptide	LecA	35^[a]^		0.5^[a]^			
			LecB	118^[a]^		536^[a]^			
**P_4_Cyc**	[106]	Cyclic β‐peptoid	LecA	296^[a]^					
**P_4_Lin**	[106]	β‐peptoid	LecA	1800^[a]^					
**PPro1**	[107]	Peptide	LecA	442^[e]^					
**PPro2**	[107]	Peptide	LecA	808^[e]^					
**PPro3**	[107]	Peptide	LecA	136^[e]^

[a] ITC; [b] ELLA; [c] Dispersal at 50 μM, unless otherwise stated; [d] HIA; [e] SPR (5 % DMSO for solubility).^[107]^

Having identified potent LecB‐inhibitors, analogous system of comparable activity against LecA were also sought, leading to identification of **GalAG2** and **GalBG2** with nanomolar‐affinity and potent biofilm‐inhibition activity (Table 5).^[53]^ **GalAG2** possessed hydrophobic aromatic aglycons and, as expected (see Section 2.1) these dendrimers had enhanced LecA‐binding compared to **GalBG2**, which possessed carboxypropyl‐β‐thiogalactoside groups instead (Figure 9). This key role of the aglycon on affinity was observed across multiple families of glycopeptide dendrimers.[^16^, ^53^, ^94^] However, the authors also point out that **GalBG2**, a higher‐valency compound with a relatively weaker K_d_, was actually a better biofilm inhibitor compared to lower‐valency but higher affinity ligands. This indicates that multivalency plays a larger role in biofilm‐inhibition than simply lectin binding constant.^[96]^

Further work attempted to optimise the peptide sequence, linkers used, as well as probing effects of multivalency, presentation, and even number of ionic residues in the sequence.^[101]^ Structural screening, wherein all amino acids in the sequence of the core scaffold (save branching lysines) were sequentially replaced by alanines, revealed little‐to‐no change in affinity and biofilm‐dispersal. Consequentially, they focused on the terminal tripeptide of dendrimer arms, in order to interactions of these with the lectin. Computational docking identified 26 tripeptides, which were synthesised as monovalent ligands for HIA screening. Two leads identified (**GalA‐KPY** and **GalA‐KRL**) both showed higher affinity in ITC than reference **GalAG0** (Section 2.1). A general trend identified was that tripeptides featuring aromatic residues at the first position had higher affinities, but this led to solubility issues when corresponding 2^nd^‐generation (G2) dendrimers were made. G2‐dendrimers, **G2KPY** and **G2KPW** had enhanced biofilm‐dispersal activity and similar affinity to **GalAG2** (Table 5). This demonstrates that amino acid sequences can be fine‐tuned to increase LecA‐affinity, particularly by having one hydrophobic and one cationic residue in the terminal tripeptide, but again that multivalency plays a much larger role in biofilm‐dispersal activity.

**FD2**’s core peptide‐sequence was employed as a scaffold for hetero‐clusters, such as **Het4G2**, which has K_d_ of 120 nM against both lectins. However homo‐dendrimers **FD2** and **GalAG2** have higher affinities for their specific lectin than hetero‐systems.^[102]^ Despite high lectin‐affinities, **Het4G2** had inferior biofilm‐dispersal and minimum biofilm‐inhibitory concentration (MBIC), while other hetero‐glycodendrimers (*e. g*. **Het2G2**) with more cationic peptide sequences were comparable to the parent homo‐dendrimers. This counterintuitive result prompted investigation into the activity of non‐glycosylated dendrimers, finding that highly cationic peptides have inherently bactericidal effects comparable to those of antibiotics polymycin‐B and tobramycin, whereas most glycosylated dendrimers, particularly those with more neutral sequences were non‐toxic to bacteria even at relatively high concentrations. Thus the mechanism of biofilm‐inhibition for cationic dendrimers involves toxicity, whereas biofilm‐inhibition by neutral compounds (*e. g*. **Het4G2** and **FD2)** is dependent on selective carbohydrate‐lectin interactions. Synergistic addition of sub‐inhibitory doses of both **FD2** and tobramycin can be used to achieve similar anti‐biofilm results as with the full dose of each individual component, thereby lowering the *de facto* dosage of each. This effect was also observed with **FucC_6_G2** (Figure 9). This may be seen because biofilm‐dispersal by non‐toxic dendrimers facilitates entry of the toxic antibiotic into cells. Similar effects were observed combining a non‐toxic and a toxic dendrimer.

Lewis^a^ is natural high‐affinity ligand for LecB, and analogue **Le^a^C_6_G2**, functionalised with this glycan indeed had heightened affinity, but lower biofilm‐inhibition than **FD2** (Table 5).^[102]^ This again underscores that higher‐affinity ligands do not directly correlate with good anti‐biofilm activity, and that structural consideration are also at play; fucoside analogue **FucC_6_G2**, showed improved biofilm‐inhibition properties.

Synthesis of higher‐generation dendrimers by direct SSPS was hindered by steric crowding. Despite low isolated yields of **2G3** and analogues, these octavalent systems demonstrated 55‐fold increase in r.p./n.^[98]^ Convergent synthesis was used to more efficiently make 3^rd^‐generation analogues of **GalAG2** and **GalBG2** to probe any multivalency enhancements on LecA‐binding.^[103]^ In terms of r.p./n, gains are not dramatic on expanding from 2^nd^‐ to 3^rd^‐generation, while higher generations proved detrimental. Furthermore solubility issues and formation of precipitates hampered ITC. However, octavalent **GalAxG3** did show modestly higher biofilm‐inhibition and ‐dispersal than tetravalent **GalAG2**.

XRD of tetravalent fucoclusters with LecB demonstrated cross‐linking of two tetramers,^[103]^ while the structure of octavalent **GalAxG3PS** shows aggregative chelate binding with LecA (Figure 5c): all 8 epitopes were bound to different LecA tetramers creating a checker‐board pattern, rationalising the tendencies of higher‐generation dendrimers to form precipitates in ITC.

Renaudet and co‐workers expanded peptide‐scaffold strategies to include their ‘RAFT’ cyclopeptide scaffolds^[108]^ in addition to lysine‐based dendrimers to obtain multivalent clusters.^[104]^ Tetra‐, hexa‐ and hexadecavalent scaffolds were synthesised by efficient oxime conjugation in all possible combinations of cyclopeptide and peptide dendrimer components (*e. g*. **RD_16_** and **RR_16_**). Tetravalent presentation of α‐fucosides (**R_4_‐αFuc**) did not lead to significant enhancement by the multivalent glycocluster effect, with increases simply mirroring concentration effects. For the hexadecavalent compounds, **RD_16_‐αFuc** with the rigid cyclopeptide core and more flexible lysine‐dendron arms was found to best enhance binding potency, with r.p./n of 64.6 (contrasting with only 5 for all‐cyclopeptide structure **RR_16_‐αFuc**), pointing to more favourable epitope spatial arrangements. This illustrates that structural parameters such as orientation and distribution can be more important than simple multivalency effects. Similar enhancements were also seen for hexadecavalent β‐fucosides, *e. g*. **RD_16_‐βFuc**, but none outcompeted α‐fucoside analogues (IC_50_ 51 to 109 nM vs 0.6 to 11 nM). This is in keeping with the known lectin selectivity. ITC of both **RD_16_‐αFuc** and **RD_16_‐βFuc** revealed lectin‐binding was enthalpy‐driven, with strong entropic barriers in both cases. The α‐fucoside has strong binding‐enthalpy (ΔH°=−223 kcal/mol) arising from this anomer better fitting the binding‐site. As such, **RD_16_‐αFuc** has K_d_ tenfold higher than the β‐fucoside. Stoichiometric information from ITC points to interaction with only 3–6 monomers of LecB by glycoclusters, suggesting not all 16 sugars are involved. An aggregative chelate binding mode was proposed.

In addition to oxime ligation, CuAAC was also used to functionalise cyclopeptide scaffolds.^[105]^ Comparison of tetravalent galactoclusters **4Gal_trz_** and **4Gal_ox_** showed significant enhancements in LecA binding (K_d_ 22 vs 91, Table 5), with triazolyl‐derivatives having a remarkable r.p./n of 1705, consistent with the established binding benefits of aromatic aglycons.^[57]^ Orthogonal use of oxime ligation and CuAAC produced a library of related hexadecavalent hetero‐glycoclusters with combinations of fucosides, mannosides and galactosides, *e. g*. **HGC1** and **HGC2**. These mixed compounds, containing a mix of α‐fucoside and β‐galactoside, retain nanomolar affinity for both LecA and LecB, and the presence of and crowding by the non‐specific epitope doesn't detrimentally effect binding affinities, when compared with homo‐glycocluster analogues.^[105]^

In a related, but simplified approach, β‐peptoids were also used as scaffolds for LecA‐targeting by Cecioni *et* *al*.^[106]^ Both linear and cyclic tetravalent β‐peptoids, Figure 9c, showed low‐micromolar activity. Cyclic scaffold presentation seen in **P_4_Cyc** had higher affinity than the linear equivalent **P_4_Lin**, with a 25‐fold increase in r.p./n by SPR, and 500‐fold by ITC. (Table 5). Cyclic scaffolds were less sterically constrained, allowing all four epitopes to interact with LecA binding‐sites.

Wang and co‐workers used the rigid helical polyproline structure as a scaffold to control the spacing and presentation of galactosides. Initially, a microarray was prepared by immobilising various functionalised polyproline peptide helices on a fluorous surface to assess LecA‐interactions.^[109]^ Each three‐residue turn of this peptide helix spaces a conjugated functional group by multiples of 9 Å on the same face of the scaffold. Galactoside‐derivatives, synthesised by SSPS and CuAAC, with spacing of 9, 18 and 27 Å between glycans were immobilised and their binding with fluorescently tagged LecA was studied. At an appropriate surface‐distribution, fluorescence binding data clearly suggested that glycopeptides can differentiate the spatial specificity of LecA, with the 27 Å spacing (K_d,surface_=354 nM) being very well matched to the known distance between LecA galactose‐binding sites.^[71]^

Compound **PProA**, Figure 9c, was analogous to the fluorous‐immobilised peptide and in SPR experiments with LecA showed K_d_ of 442 nM (compared to 808 nM for shorter spaced **PPro2**, Table 5), confirming that appropriately‐spaced epitopes gave similar binding‐enhancement in solution. As expected, introduction of aromatic aglycons into these structures (**PPro3**) further improved binding affinity.^[107]^

### 3.3 Other dendrimers

In addition to glycopeptide dendrimers, other dendrimer classes are also reported as PA lectin‐inhibitors.^[110]^ A library of glycodendrimers synthesised by a convergent approach using CuAAC included **DenAFuc** and **DenAFucGal**.^[111]^ Turbidimetric assays showed rapid formation of insoluble complexes, indicating cross‐linking with LecB (for fucodendrimer) and both lectins (for hetero‐dendrimer). No quantitative lectin‐binding assessments were reported.

Another class of dendrimer was supramolecularly self‐assembled from amphiphilic glycoconjugates possessing azo‐benzene hydrophobic tail groups, which have capacity for light‐activated *trans‐cis* isomerisation.^[112]^ Amphiphiles (*e. g*. **DenB**) aggregated into cylindrical micelles with effective diameters ∼100 nm (DLS). Lectin‐inhibition was assessed by competitive FP binding assay and results were disappointing, with large degrees of multivalency (hundreds of sugars per dendrimer) leading to no sizable enhancement of micromolar IC_50_, when compared to monosaccharide references. This is perhaps due to excessive flexibility in dendrimer structure. Manno‐dendrimers, however, did show 30–80‐fold binding‐enhancement compared to Me‐αMan (IC_50_ 2.7 μM for LecB). Unfortunately, no significant photomodulation was seen for either dendrimer aggregation or lectin‐inhibition, likely due to low photo‐isomerisation yields (perhaps owing to densely packed aggregate geometry impeding micelle light penetration).

The first use of Saturation Transfer Difference‐NMR to assess LecA‐ligand interactions was reported for 18‐valent galacto‐dendrimer **DenGal_18_**, showing both Gal epitopes and dendrimer scaffold interacting with LecA in solution (K_d_ 41 μM). Man‐ or non‐glycosylated analogues demonstrated no lectin interactions. **DenGal_18_** inhibited PAO1 biofilm‐formation *in* *vitro*, giving a 1.8‐fold decrease in biofilm mass at 250 μM.^[113]^

### 3.4 Aromatic macrocycle scaffolds

**Figure 10:**
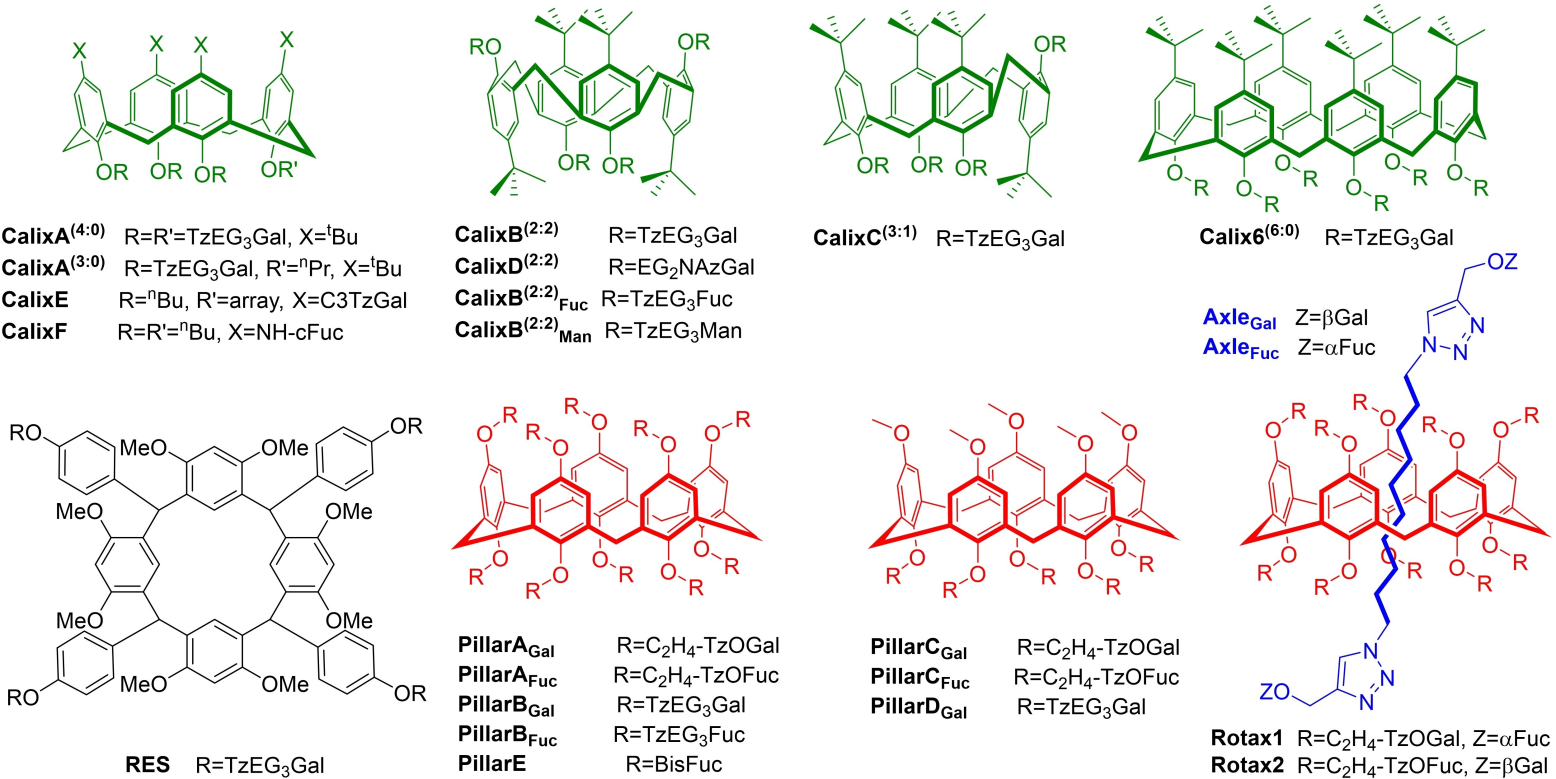
Selected macrocyclic and supramolecular scaffolds for glycoclusters.

Macrocycles, including calixarenes and others (Figure 10), are attractive scaffolds for lectin‐targeting mainly due to their versatility in achieving varying topologies and valencies,^[114]^ and several glycocluster families based on macrocylic cores are reported.

Imberty, Matthews and Vidal reported what would be the first in a series of papers exploring calix[4]arene scaffolds, **CalixA‐C**, as PA lectin‐inhibitors.^[75]^ In this seminal study, effects of varying presentation‐topologies of carbohydrates were assessed. Varying degrees of propargylation on either the macrocycle's lower rim or both rims allowed EG_3_‐linked galacto‐ and mannosides to be coupled to scaffolds by CuAAC. The standard “cone” calix[4]arene geometry was utilised, as well as the 1,3‐alternate conformer to explore the relevance of differing topologies, in addition to multivalency. ITC with LecA determined that trivalent cluster **CalixA^(3:0)^** only has micromolar affinity, while all tetravalent examples had submicromolar affinity of 176–420 nM (Table 6), with at least double the enthalpic contribution to monovalent analogues. Strong differences were observed in the thermodynamic behaviour of tetravalent clusters, dependent on “arm” topology. Partial inversion of the “cone” conformers in **CalixB^(2:2)^** and **CalixC^(3:1)^** leads to 2‐fold K_d_ enhancement, with the “2 : 2” presentation (**CalixB^(2:2)^**, Figure 10) being the most potent inhibitor (K_d_=176 nM, r.p./n 144). Observed stoichiometry of **CalixB^(2:2)^** (*n=*0.26) indicated that it could chelate two binding‐sites on one LecA tetramer as well as two further sites on a second tetramer, leading to aggregative‐chelate complexes (Figure 5b). This hypothesis was supported by AFM studies where an equilibrated solution of **CalixB^(2:2)^** and LecA was deposited on a mica surface and resulting monodimensional filament structures formed, matching the model and dimensions of aggregative chelate binding, with occasional defects leading to branching in the self‐assembly structure. This is a powerful tool for more precisely describing glycocluster binding mode.^[115]^

**Table 6:** Affinity of selected macrocyclic glycoclusters.

Compound	Ref.	Scaffold	Lectin	K_d_ [nM]	IC_50_ [nM]	r.p./n
**CalixA^(3:0)^**	[75]	Calix[4]arene	LecA	2050^[a]^	6400^[b]^	24^[a]^
**CalixA^(4:0)^**	[75]	Calix[4]arene	LecA	420^[a]^	2500^[b]^	89^[a]^
**CalixB^(2:2)^**	[75]	Calix[4]arene	LecA	176^[a]^	500^[b]^	213^[a]^
**CalixC^(3:1)^**	[75]	Calix[4]arene	LecA	200^[a]^	1700^[b]^	188^[a]^
**CalixD^(2:2)^**	[57]	Calix[4]arene	LecA	90^[a]^	1000^[b]^	284^[a]^
**Calix6^(6:0)^**	[106]	Calix[6]arene	LecA	140^[a]^	800^[b]^	179^[a]^
**CalixB^(2:2)^**_**Fuc**_	[118]	Calix[4]arene	LecB	48^[a]^		1.5^[a]^
**CalixB^(2:2)^**_**Man**_	[118]	Calix[4]arene	LecB	2267^[a]^		
**RES**	[119]	Rescorcinarene	LecA		700^[c]^	78.5^[c]^
**PillarA_Gal_**	[120]	Pillar[5]ene	LecA	413^[a]^	26000^[c]^	16.9^[a]^
**PillarB_Gal_**	[120]	Pillar[5]ene	LecA	366^[a]^	218000^[c]^	19.1^[a]^
**PillarC_Gal_**	[121]	Pillar[5]ene	LecA	931^[a]^	29000^[c]^	15^[a]^
**PillarD_Gal_**	[121]	Pillar[5]ene	LecA	586^[a]^	9000^[c]^	23.8^[a]^
**PillarA_Fuc_**	[120]	Pillar[5]ene	LecB	990^[a]^	90^[c]^	0.04^[a]^
**PillarB_Fuc_**	[120]	Pillar[5]ene	LecB	220^[a]^	30^[c]^	0.19^[a]^
**PillarC_Fuc_**	[121]	Pillar[5]ene	LecB	1402^[a]^		0.06^[a]^
**PillarE_Fuc_**	[120]	Pillar[5]ene	LecB	150^[a]^	6^[c]^	0.29^[a]^
**Axle_Gal_**	[122]	Divalent axle	LecA	158^[a]^		
**Axle_Fuc_**	[122]	Divalent axle	LecB	112^[a]^		
**Rotax1**	[122]	Pillar[5]ene	LecA	261^[a]^		
		rotaxane	LecB	279^[a]^		
**Rotax2**	[122]	Pillar[5]ene	LecA	5360^[a]^		
		rotaxane	LecB	625^[a]^		
**CalixG_Fuc_**	[123]	Calix[4]arene	LecB		249^[d]^	0.8^[d]^
**CalixG_Man_**	[123]	Calix[4]arene	LecB		664^[d]^	59^[d]^
Rotax3	[11]	Pillar[5]ene	LecA	226^[a]^		24^[a]^
		rotaxane	LecB	171^[a]^		0.63^[a]^

[a] ITC; [b] SPR; [c] ELLA; [d] FP assay.

In direct comparisons with **P_4_Cyc** and **ZnPor** (Sections 3.2 and 3.6, respectively), all of which had TzEG_3_Gal arms, calixarene‐derivatives like **CalixB^(2:2)^** had higher LecA‐affinity.^[106]^ Hexavalent **Calix6^(6:0)^** performed even better, and a “bind and jump” mechanism for its interaction with LecA tetramers was proposed to explain the 1 : 3 stoichiometry calculated from ITC.

**CalixB^(2:2)^** analogues with five different linker‐arms were designed to explore variations in length, rigidity and hydrophobicity on tetravalent scaffolds.^[57]^ Analysing arm‐precursors as monovalent ligands, **GalPhNAz‐N_3_** was by far the best inhibitor (K_d_ 5.8 μM), as expected, with the presence of aromatic aglycons giving stronger enthalpy of binding. However, due to the added hydrophobicity and rigidity of the linker, this arm led to major solubility issues when coupled with nearly all scaffolds studied, resulting in limited data from biological assays. Even against a larger library, **CalixB^(2:2)^** continues to outperform other structures in SPR, including its analogue with EG_2_NAz‐ linker **CalixD^(2:2)^** (Table 6). This study illustrated very well the importance of balancing increased potency (which hydrophobic aglycons can provide) with solubility, linker‐length and presentation topology. **CalixB^(2:2)^** seems to combine these elements in a serendipitous way. It strikes the balance to excel in all measures (HIA, ELLA, SPR and ITC) both compared against its own derivatives, other calix[4]arene topologies and also entirely different scaffolds.

Calix[4]arenes functionalised at the upper rim, **CalixE**, were immobilised onto oligonucleotide scaffolds (discussed in Section 3.1) to assess their LecA‐recognition.^[116]^ Unexpectedly, these were not found to bind Alexa 647‐labelled LecA, in contrast to trivalent linear presentation modes, potentially due to steric crowding. This however does not correlate well to the excellent results seen with lower‐rim decorated tetravalent **CalixA^(4:0)^**.

The first fucosylated calix[4]arene ligand to target LecB, **CalixF**, displayed four acetylglycylamido‐linked epitopes on the macrocycle's upper rim, and this showed biofilm‐inhibition of up to 80 % at 200 μM. This behaviour was dose‐dependent and **CalixF** biofilm‐inhibition always exceeded the activity of a non‐fucosylated analogue. However, modest activity of this analogue points to possible non‐specific anti‐biofilm interactions in addition to lectin binding.^[117]^

Fucosylated analogue of **CalixB^(2:2)^**, was also found to be an excellent LecB‐inhibitor (K_d_ 48 nM).^[118]^ ITC studies of the thermodynamics of **CalixB^(2:2)^**_**Fuc**_ binding, and the unimpressive increase in r.p./n (1.5‐fold), pointed to a “bind and jump” mechanism of the lectin around the glycocluster structure. A chelate binding mode is not possible for LecB with this structure, since its binding sites are more distant from each other than in LecA (40 vs 29 Å, see Figure 1).

This multi‐team study provides a remarkably complete set of data to assess PA lectin‐inhibitors as anti‐infection agents, surpassing much that preceded it. Both **CalixB^(2:2)^** and **CalixB^(2:2)^**_**Fuc**_ were evaluated for various biological effects relevant to the virulence of PA. Their impact on bacterial cell aggregation was estimated with wild‐type PAO1, as well as PAO1*ΔlecA* and PAO1*ΔlecB* (isogenic knockout mutants not expressing LecA and LecB, respectively). At concentrations of 100 μM, both tetravalent calix[4]arene glycoclusters led to aggregation of the wild‐type bacteria, but not of knockout strains. These results and various control experiments indicate aggregation behavior is lectin‐dependent. A dose‐dependent inhibition of PA‐adhesion to A549 human epithelial cells was also seen for both glycoclusters, reaching 70 % and 90 % for galactosides and fucosides respectively. This effect was lost in knockout strains, although galactocluster **CalixB^(2:2)^** still had some activity at higher concentrations, indicating the potential presence of targets other than LecA. At concentrations of 5 mM, both calix[4]arene clusters demonstrate significant inhibition of biofilm‐formation by all three strains of PA. This result is counterintuitive, since activity is seen even in the absence of target lectins, but is specific to Gal‐ and Fuc‐derivatives, and not seen for Glc‐ and Man‐analogues. Finally, these compounds (at millimolar concentrations) were shown to be effective at protecting against bacterial lung‐injury in an *in* *vivo* mouse model. Bacterial load in lungs and spleen was also decreased in test subjects. Importantly nanomolar affinities *in* *vitro* did not translate directly to *in* *vivo* studies, with higher concentrations required, likely due to competition from other adhesins, lectins and proteins in more complex systems. To date these remain the only multivalent PA lectin ligands evaluated *in* *vivo*.^[118]^

Apart from calixarenes, other macrocyclic scaffolds have also been used, including attempts to use a porphyrin macrocycle‐scaffold for multivalent glycoconjugates through CuAAC,^[106]^ but the macrocycle sequestered copper, interfering with this strategy. Instead the Zn(II)‐porphyrin complex **ZnPor** was used to form square planar tetravalent ligands (see Section 3.7). Rescorcin[4]arenes **RES** were also prepared by CuAAC, but displayed IC_50_ values lower than calix[4]arenes and thermodynamic insight into decreased affinity couldn't be obtained due to solubility issues.^[119]^

Pillar[5]enes are applied as densely‐functionalised scaffolds, with a series of decavalent (**PillarA‐B**)^[120]^ and pentavalent (**PillarC‐D**)^[121]^ ligands, functionalised with carbohydrates with varying linkers. Difficulty separating the diasteroisomers of pillar[5]enes is an issue with this class of glycocluster, which is not yet resolved; nonetheless studies have been performed on 1 : 1 isomeric mixtures. Galactosylated pillar[5]ene clusters were strong LecA‐ligands, with increased linker‐flexibility from **PillarA_Gal_** to **PillarB_Gal_** leading to enhanced K_d_ (Table 6). Increased multivalency between decavalent and pentavalent systems, however, yielded only moderate improvements. This is rationalised through stoichiometries observed in ITC, where only five LecA tetramers interact with decavalent clusters, and thus little advantage arises from extra Gal‐epitopes. Their binding behaviour, nonetheless compared unfavourably to tetravalent **CalixB^(2:2)^**. This leads to the conclusion that for macrocyclic scaffolds, increased multivalency leads to enhanced LecA binding, but only up to a point, with linker‐length, flexibility and presentation also playing important roles.

LecB affinity of fucosylated pillar[5]enes depended on multivalency, and to a lesser extent, linker‐length. In ITC, pentavalent ligands **PillarC_Fuc_** gave disappointing millimolar affinities,^[121]^ while decavalent compounds saw a significant K_d_ increase upon addition of EG_3_‐spacers (220 nM for **PillarA_Fuc_** vs. 990 nM for **PillarB_Fuc_**, Table 6). This difference is perhaps due to steric crowding limiting optimal Fuc interactions with LecB. Extension of these structures to flexible 20‐valent cluster **PillarE_Fuc_**, with triazolyl aglycons, gave the highest affinity ligands (K_d_=150 nM), representing very potent LecB‐inhibitors.^[120]^

In an imaginative extension of this work, **PillarA** were also employed as the “wheel” component of mechanically‐interlocked [2]rotaxane molecules, where the “axle” is a divalent glycoconjugate (**Axle_Gal_ or Axle_Fuc_**), assembled by CuAAC. Sugar motifs on the axle act as “stoppers” to prevent de‐threading.^[122]^ This strategy resulted in heteroclusters **Rotax1‐2**, where axle and wheel were functionalised with alternate saccharides (β‐Gal or α‐Fuc). The divalent axle compounds and their respective [2]rotaxanes showed similar binding affinities to their target lectin, indicating that the pillar[5]ene has no dramatic negative impact on their binding. As seen already for above structures, LecA‐binding is sensitive to multivalency effects, due to chelate‐binding, and **Rotax1** has 268‐fold increase r.p./n. This [2]rotaxane can thus inhibit both LecA and LecB at similar submillimolar K_d_ values (Table 6), without the non‐specific carbohydrate impeding binding. Stoichiometries determined from ITC suggest this sophisticated system could cluster LecA tetramers and also aggregate LecB. However, these systems were not effective at biofilm‐inhibition.

### 3.5 Carbohydrate‐based macrocycles

Mazzaglia *et* *al*. report amphiphilic β‐cyclodextrin derivatives can aggregate into nanoparticles in solution. The macrocylic core was decorated with thiogalactosides, connected by oligo(ethylene glycol) spacers, and these formed particles with diameters of ∼100 nm. Their ability to bind LecA was supported by new peaks in mass spectrometry, and more detailed physical studies showed that the lectin's dynamics (as determined by light‐scattering) are slowed down upon interaction with as little as 2 equivalents of **CD**. A characteristic and persistent decrease in LecA's intrinsic fluorescence upon addition of galactosylated cyclodextrin‐derivative was measured, as well as decrease in time‐resolved fluorescence lifetimes and increase in steady‐state fluorescence anisotropy. None of these changes were observed for glucosylated control compounds.[^124^, ^125^]

**Figure 11:**
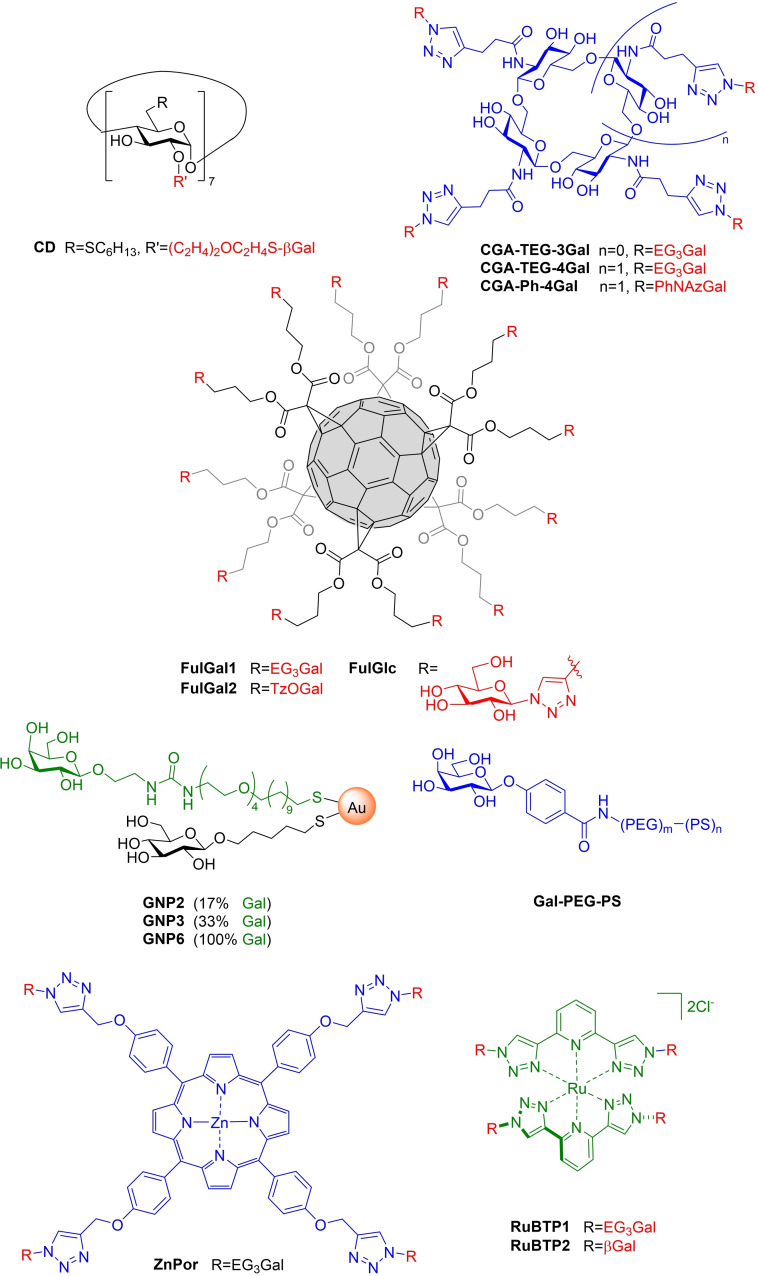
Selected structures of multivalent glycocluster carbohydrate‐based macrocycles, nanomaterials and metal complexes.

Cyclic oligo‐(1→6)‐β‐D‐glucosamine scaffolds were used for 15 glycoclusters with various valencies and linkers, including examples **CGA** (Figure 11).^[126]^ Increasing multivalency of compounds with flexible EG_3_‐linkers, *e. g*. from **CGA‐TEG‐3Gal** to **CGA‐TEG‐4Gal**, led to increases in binding‐affinity as expected, however, the r.p./n gain was not noteworthy (Table 7). Significant affinity enhancement was achieved by using rigid hydrophobic phenyltriazole‐linkers instead. A marked advantage of this scaffold over calixarene scaffolds (Section 3.4) is that they do not present solubility issues when functionalised with epitopes featuring phenyltriazole linkers.^[57]^ Best results were obtained with tetravalent galactosides cluster **CGA‐Ph‐4Gal**. With K_d_ of 79 nM, it was among the best ligands tested to that point (by ITC, ELLA and HIA). This indicates that these macrocylic carbohydrate‐derived scaffolds present a versatile and effective platform for glycocluster design.

**Table 7:** Affinity of selected compounds from Sections 3.5–7.

Compound	Ref.	Scaffold	Lectin	K_d_ [nM]^[a]^	IC_50_ [nM]	r.p./n	HIA MIC [μM]
CGA‐TEG‐3Gal	[126]	Cyclic glucosamine	LecA	460	270^[b]^	50^[a]^ (63^[b]^)	98
CGA‐TEG‐4Gal	[126]	Cyclic glucosamine	LecA	310	150^[b]^	56^[a]^ (69^[b]^)	49
CGA−Ph‐4Gal	[126]	Cyclic glucosamine	LecA	79	57^[b]^	222^[a]^ (303^[b]^)	1.2
FulGal1	[127]	Fullerene	LecA		688^[b]^	27^[b]^	250 (3^[d]^)
FulGal2	[127]	Fullerene	LecA		40^[b]^	458^[b]^	0.78 (1068^[d]^)
FulGlc	[127]	Fullerene	LecA		233×10^3[b]^	0.08^[b]^	63
GNP2	[128]	AuNP	LecA	5800		2^[a]^	
GNP3	[128]	AuNP	LecA	760		12^[a]^	
GNP6	[128]	AuNP	LecA	50		42^[a]^	
100 %‐GalNP	[129]	Polymer NP	LecA				6.31 (495^[d]^)
50 %‐GalNP	[129]	Polymer NP	LecA				3.15 (992^[d]^)
ZnPor	[106]	Zn‐porphyrin	LecA	332	500^[b]^ (1400^[c]^)	113.5^[a]^	63 (159^[d]^)

[a] ITC; [b] ELLA; [c] SPR; [d] relative potency by HIA.

### 3.6 Nanomaterials and nanoparticles

Nanomaterials and ‐particles present potential as platforms for highly‐multivalent glycoclusters. This has not been ignored by researchers targeting lectins, exploiting their potential to provide glycocalyx‐like surfaces for presenting carbohydrates.^[128]^ So‐called “fullerene sugar‐balls” were synthesised using CuAAC chemistry to graft unprotected sugar‐derivatives onto alkyne‐ or azide‐functionalised C_60_. This yields glycoclusters with globular topology, where carbohydrate epitopes are almost equidistant with overall tetrahedral symmetry.^[130]^ Such systems were studied as carbohydrate‐processing enzyme inhibitors^[131]^ and FimH inhibitors,^[132]^ before their interactions with LecA were studied.^[127]^ Dodecavalent fullerenes **FulGal1** and **FulGal2** had nanomolar IC_50_ for LecA (Table 7), with potency enhanced by the “glycoside cluster effect”. Higher affinity of the latter was rationalised in terms of the aromatic Tz group adjacent to the galactose, available for interactions with hydrophobic residues in LecA's binding site. An unexpected result was obtained with a Glc analogue of these systems, **FulGlc**, acting as a modest inhibitor for LecA, on a similar scale to the monosaccharide reference, indicating the possibility of additional mechanisms for “sugar‐balls” to inhibit LecA even through non‐specific binding.

Gold nanoparticles with a core diameter of <2 nm were functionalised with thiol‐containing glycoconjugates. Different presentation densities were assessed, by adding varying ratios of galactoside and glucoside arms (Figure 11) to give **GNP2**, **GNP3** and **GNP6** (among others) with galactose presentation densities of 17 %, 33 % and 100 %, and valencies of 12, 15 and 67 respectively.^[128]^ Interactions with LecA were studied by qualitative HIA, and quantitatively by SPR and ITC. All three techniques showed similar trends, with ligands presented on nanoparticles demonstrating increases in avidity for LecA, compared to free glycoconjugates in solution. This further increased with presentation density; **GNP6** had nearly a 3000‐fold affinity enhancement (K_d_ 50 nM, Table 7). ITC pointed to reduction in entropic penalties being key to enhanced binding, with presentation density important for increasing enthalpic contributions. Increase in ligand‐activity was explained by both increased effective concentration of Gal, as well as structural complementarities, such as increasing the likelihood of statistical rebinding to a ligand and increased ligand‐binding site overlap. This multivalent nanoparticle system presents a promising platform for further development but has not yet been followed up with antiadhesive studies.

Biological assays, however, are reported for surface‐modified polymer nanoparticles, based on polystyrene‐polyethyleneglycol co‐polymers.^[129]^ Nanoparticles with diameters of ∼80 nm were prepared by flash nanoprecipitation and contained different ratios of unmodified polymer and galactoside‐modified polymer **Gal‐PEG‐PS**. In HIA, inhibition was observed for the **50** **%**‐ and **100** **%‐GalNP** nanoparticles, but not 25 %‐modified systems, indicating an optimum form of presentation with appropriate local Gal concentration, but that the 100 %‐modified nanoparticle may result in steric inhibition of lectin‐binding. These two systems inhibited biofilm‐formation at Gal concentrations above 6.3 and 12.6 μM, respectively. Crystal‐violet assay and confocal microscopy was used to assess this behaviour, and it was confirmed that the nanoparticles do not inhibit bacterial growth at the concentrations tested, and these systems are antivirulent, rather than antibiotic. The authors point out that potential future applications of these structures could rely on encapsulating fluorescent or drug molecule in lipophilic core for targeted therapeutic or diagnostic treatments.

### 3.7 Metal complexes

Among the large variety of multivalent scaffolds, the use of metal complexes in this field is very rare, with only two examples to the best of our knowledge. A propargylated porphyrin scaffold was selected by Cecioni *et* *al*. for comparison with **CalixB^(2:2)^** and **P_4_Cyc**, but CuAAC reactions were unsuccessful for the unmetallated scaffold (due to copper‐complexation). Consequently, the scaffold was complexed with Zn(II) before successful triazole formation, giving **ZnPor** (Figure 11), a tetravalent galactocluster with flexible EG_3_‐arms.^[106]^ Here the Zn(II) ion has no structural or functional role, but seems to be present only for synthetic simplicity. **ZnPor** is a more potent HIA inhibitor for LecA than either calix[4]arene or β‐peptoid derivatives in the same study, owing perhaps to its square planar geometry. Similar evidence of LecA‐affinity was seen in SPR. ITC measurements showed that **ZnPor** had a K_d_ of 332 nM and the rigid and planar topology seems to be conducive to lowering entropy costs of binding, allowing for 1 : 2 complexes to form with the lectin (but not for all four epitopes to engage in aggregative‐chelate binding).[^57^, ^106^]

Byrne and co‐workers recently reported use of Ru(II)‐coordination chemistry to template formation of metal‐centred tetravalent glycoclusters from lower valency ligands, namely divalent bis(triazolyl)pyridine glycoconjugates.[^133^, ^134^] Clusters based on various carbohydrate epitopes were tested for their ability to inhibit PAO1 biofilm‐formation. Tetravalent galactocluster **RuBTP1** with flexible EG_3_‐spacers inhibited biofilm‐formation at 5 mM, while neither the precursor ligand, nor complex **RuBTP2** (without spacer) did. None of the Ru(II)‐complexes tested were bactericidal or bacteriostatic, discounting an active therapeutic role for the metal in this anti‐biofilm activity. Instead, the coordination geometry and appropriate linker‐length is proposed to be favourable for chelating adjacent Gal binding‐sites in LecA, as was seen for calix[4]arene structures of similar geometry, **CalixB^(2:2)^**.^[118]^ Use of metal ions of different coordination geometry to tune the activity of multivalent glycoclusters presents opportunities for future developments.

## 4 Coupling Lectin Targeting with Additional Functionality

**Figure 12:**
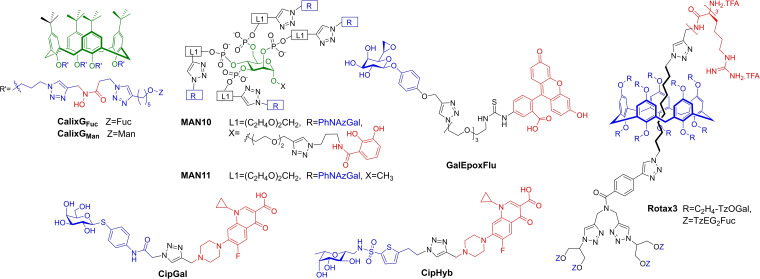
Selected examples of glycoclusters coupled with additional functionality. Lectin‐targeting components are shown in blue, and active components in red.

Among the most inspiring recent examples of PA lectin inhibitors are those which couple this strategy with further innovative functionality, in search of therapeutic or diagnostic tools. Some complimentary anti‐virulence, imaging and antimicrobial examples are highlighted here (Figure 12), along with their future outlook.

Two different approaches have been reported, attempting to simultaneously target lectins and hijack PA's siderophore pathway, which uses iron‐chelators to transport Fe(III) to the cell membrane for metabolic activity. In addition to siderophores produced by the bacterium, PA can recruit exogenous siderophores. Calix[4]arene‐clusters **CalixG** (Figure 12, Table 7), with hydroxamic acid arms, put PAO1 cells under stress under iron‐limiting incubation conditions, causing increased production of endogenous fluorescent siderophore pyoverdine‐I, as PA competes with chelating **CalixG** for Fe(III).^[123]^ Moreover bacterial growth of a siderophore‐deficient PA strain was significantly disrupted by **CalixG_Man_**, and to a weaker extent by **CalixG_Fuc_**, indicating that calix[4]arenes were not recruited as exogenous iron‐chelators. Biofilm‐inhibition results of these systems, however, were not as expected, with fucosides being inactive but **CalixG_Man_** giving reduction of 70 % at 50 μM. Curiously, a tested α‐glucocluster analogue, demonstrated very strong biofilm‐inhibition (92 % at 20 μM). This raises serious questions about whether lectin‐binding is key to anti‐biofilm effects, since glucose does not interact with LecB. Possibly, the hydroxamic acid arms play a more significant role, either due to anionic charge or their propensity to release nitric oxide, which is known inhibit biofilms.^[135]^ This study highlights the importance of adequate control experiments before antipseudomonal activity is unambiguously attributed to lectin‐inhibition.

Carbohydrate‐centred clusters derived from **MAN4** (Section 3.1) functionalised to include catechol or hydroxamate arms for iron‐chelation were also studied with PAO1 (and several knockout strains), indicating that catechol‐appended galactoclusers penetrated the bacterial envelope, exploiting the siderophore pathway in a “Trojan horse” strategy.^[136]^ Despite evidence for localisation at the cell membrane (where 5 % of LecA is located), no decrease in virulence was observed. Protection assays of human pulmonary cell cultures against PA demonstrate up to 70 % protection with galactocluster–siderophore conjugates (*e. g*. **MAN10**), but disappointingly, this was similar to catechol‐free analogues. While neither of these examples effectively exploit the potential of simultaneously targeting two virulence pathways, both provide important insights into overlaps of these areas and it is possible that further work will identify different lectin‐ligands, which will benefit from additional targeting *vi*a the siderophore pathway.

Epoxide‐functionalised galactomimetic **GalEpoxFlu**, conjugated with fluorescein demonstrates a useful application of covalent lectin‐inhibition, whereby LecA can be used as a target to visualise PA biofilms. Biofilms were grown with PAO1 and a *ΔlecA* knockout*‐*mutant, with subsequent addition of **GalEpoxFlu**. Under confocal microscopy, specific staining of wildtype aggregates was obvious, and was not seen for *ΔlecA* biofilms. This has potential in diagnostics for PA, or as an imaging approach for directed therapies.^[59]^

Titz and co‐workers recently reported an innovative strategy for lectin‐targeted delivery of antibiotics, where high potency lectin probes (Section 2.1) were conjugated to ciprofloxacin.^[12]^ Binding‐affinity of conjugates for LecA and LecB didn't differ significantly from the parent glycosides, and **CipHyb** (Figure 12) effectively bound LecB from both PAO1 and PA14 strains. Encouragingly, accumulation of these conjugates in the biofilm was higher than ciprofloxacin alone, corresponding with increased concentration of lectins in this difficult‐to‐drug environment. This observation is key to supporting the hypothesis that lectin‐targeting allows conjugates to further penetrate biofilm. Unfortunately both strains of PA showed lower antimicrobial‐susceptibility to conjugates than to ciprofloxacin. Decreased antibiotic activity is also seen upon initial structural modification of the antibiotic and so this alkylation step may be the key indicator of decreased activity. Nevertheless, this strategy doesn't lack potential, and further development should be pursued.

In recent developments on [2]rotaxanes (**Rotax2**, Figure 10), introduction of polycationic guanidinium‐containing polypeptide “potency modules” into the structure led to significant biofilm‐inhibition and dispersal against PAO1. One example, **Rotax3** (Figure 12, Table 6), gave >50 % biofilm inhibition at a MBIC_50_ of 2.4 μM. Such anti‐biofilm activity was previously shown for other bacteria but suffered from a lack of specificity. This heteroglycocluster leads to targeted effects for PA; anti‐biofilm activity is not seen when the *ΔlecAΔlecB* knockout strain was used, nor for *Staphylococcus aureus*. This confirms that activity is driven by lectin‐binding. These rotaxane anti‐virulence compounds were non‐bactericidal and were found not to damage red blood cell membranes. These impressive results marry lectin‐targeting with separate anti‐virulence effects and offers much scope for additional development.^[11]^ The future of this field will rely on translation of lectin‐targeting to agents, like those outlined in this section, to be effective in a clinical setting.

## 5 Conclusions

The main considerations which have been shown to impact inhibitor affinity and effectiveness for anti‐biofilm and antiadhesive effects are: **(a)** the correct choice of carbohydrate epitope; **(b)** the role of linker length, hydrophobicity and rigidity; and **(c)** carbohydrate presentation and multivalency (scaffold topology).

- Native monosaccharide ligands for PA lectins serve as a reliable starting point for building more sophisticated higher‐affinity glycoconjugate inhibitors. For LecA, almost all reported examples are based on d‐Gal epitopes. For LecB, the situation is slightly more complex. Many fucosides and fucoclusters are reported with high affinities for LecB, and indeed LecB has unusually high affinity for l‐Fuc, by comparison to other C‐type lectins. However it is less selective, as also recognises d‐Man (with a similar affinity to LecA's binding with d‐Gal) and d‐arabinose. The challenge of selective targeting has been addressed to give molecules with drug‐like properties, by incremental rational design of bespoke glycomimetics (Section 2.1), combining structural aspects of d‐Man and l‐Fuc which promote strong protein‐ligand interactions, giving selectivity and affinities comparable to natural glycans.
- LecA‐inhibition benefits significantly from careful tuning of the properties of the linker between several epitopes due to two key structural factors. Firstly, the His50 residue in LecA's binding pocket can engage in “T‐shaped” CH⋅⋅⋅π interactions: suitably‐positioned hydrophobic aromatic aglycons (particularly phenyl‐derivatives), greatly enhance affinity in numerous examples detailed above. Hydrophobic linkers however, in several cases lead to solubility limitations contrasting with more flexible hydrophilic EG_3_‐linkers, meaning this consideration must be balanced with other structural features of the inhibitor. Secondly, the distance between adjacent binding‐sites (∼29 Å) allows for careful design of linker‐length and rigidity to result in strong chelate‐binding. Combining these considerations has led to divalent systems with low‐nanomolar affinities. For LecB, which has a longer inter‐binding site distance, the impact of linker‐tuning is less dramatic. The ability to cross‐link two tetramers of LecB in an aggregative mode plays a greater role, and a chelate‐binding mode is seldom proposed.
- Multivalency increases avidity for many inhibitor‐lectin interactions, but LecA‐targeting systems benefit most markedly from glycoside cluster effects, resulting in enhanced potency per epitope beyond simple concentration effects. Frequently this has been shown, by various powerful analytical techniques, to result from combinations of chelate and/or aggregative binding‐modes, which are possible when several LecA tetramers can be cross‐linked. Many scaffold topologies have been explored, including carbohydrate‐scaffolds, peptide‐based and non‐peptide dendrimers, macrocycles, nanomaterials and metal complexes. No scaffold has a monopoly on successful lectin‐binding with potent examples reported in each category. Specific scaffold geometries have been shown to have significant effects, even among otherwise analogous structures. Prime examples include calix[4]arene, mannose‐centred and glycopeptide dendrimer clusters, where optimum topologies were found through iterative modifications, often favouring epitope presentation suitable for enhanced cross‐linking of lectins.

The most promising avenue for therapeutic developments in this field is biofilm‐inhibition and/or dispersal, including drug‐delivery to infection sites using PA's lectins as targets. This approach, and anti‐adhesive strategies, which aid the clearance of infection, or decrease the physical barrier biofilms present to antibiotics, would have great value in tackling PA in vulnerable patients. Non‐bactericidal anti‐biofilm agents are particularly highlighted, since they evade the risk of PA evolving resistance to such therapeutics. Monovalent glycomimetics for LecB have demonstrated biofilm‐inhibition. Tetravalent galactoclusters with epitopes suitably placed to cross‐link several lectin tetramers are well‐represented among anti‐biofilm candidates (*e*. *g*. **Bis(U3Ph’)**, **MAN5‐a**, **GalAG2**, **CalixB^(2:2)^**, **RuBTP1**), highlighting the significant role multivalent scaffold topology can play in antibiofilm activity. Glycopeptide dendrimers, targeting both lectins, are the best‐represented class of ligand, where biofilm‐inhibition and dispersal of biofilms was studied, with significant activity for both at micromolar concentrations *in* *vitro*. They also highlighted the importance of tuning structure, since polycationic dendrimers had bactericidal anti‐biofilm effects, which were not lectin‐targeted, by contrast to neutral structures with targeted effects. Use of knockout PA strains which do not produce lectins is key to correctly attributing activity to lectin‐inhibition. In several reported cases, the exact origin of biofilm‐inhibition remains unclear. Noticeably, high binding‐affinity is not directly correlated with anti‐biofilm activity and the role of topology and binding‐mode cannot be underestimated. Indeed, despite excellent nanomolar affinities, calix[4]arene systems required millimolar doses to inhibit biofilm. To date these studies have some of the most advanced sets of biological testing for multivalent PA lectin‐inhibitors, with anti‐adhesive tests, biofilm‐inhibition and ‐dispersal, and an *in* *vivo* mouse model of lung‐injury.

As consensus emerges on the criteria required for effective inhibitor design for LecA and LecB, in terms of advantageous binding pocket interactions, rigidity and cross‐linking ability of the ligand (see points **(a)‐(c)** above), development of tightly‐binding inhibitors has become more straightforward, allowing more ambitious applications to be explored. Recent advances that carefully apply some or all of these ligand structure design criteria show the localization of antibiotic conjugates in biofilms (but no biofilm‐inhibition), and targeted anti‐biofilm activity of heteroglycocluster [2]rotaxanes at low‐micromolar concentration. These examples represent exciting steps forward and we anticipate increased development in the near future for applications of well‐designed PA lectin‐targeting molecules in medicinal chemistry.

## Abbreviations

ADMET: absorption, distribution, metabolism, excretion and toxicity
CF: cystic fibrosis
CuAAC: copper(I)‐catalysed azide‐alkyne cycloaddition
Cy3: cyanine3 dye
DDI: DNA‐directed immobilisation
Gal: d‐galactose
Glc: d‐glucose
Fuc: l‐fucose
Man: d‐mannose
ITC: isothermal titration calorimetry
HIA: hemagglutination inhibition assay
EG: ethylene glycol
ELLA: enzyme‐linked lectin assay
FP: fluorescence polarisation
H‐bonding: hydrogen‐bonding
MBIC: minimum biofilm‐inhibition concentration
MIC: minimum inhibitory concentration
PrOF‐NMR: protein‐observed fluorine nuclear magnetic resonance
PA: *Pseudomonas aeruginosa*
r.p./n: relative potency per carbohydrate epitope (compared to a monovalent control compound)
SAR: structure‐activity relationship
SPR: surface plasmon resonance
Tz: triazole
XRD: X‐ray diffraction

## Conflict of interest

The authors declare no conflict of interest.

## Biographical Information

*Karolina Wojtczak is a PhD candidate under the supervision of Dr. Joseph Byrne in the School of Biological and Chemical Sciences at NUI Galway. She obtained her B.Sc. in Chemistry from Maynooth University in 2019 and started her PhD program that same year. She is currently researching the development of lanthanide‐based luminescent glycoconjugates as sensors targeting bacterial lectins, with an application in novel rapid diagnostic devices*.

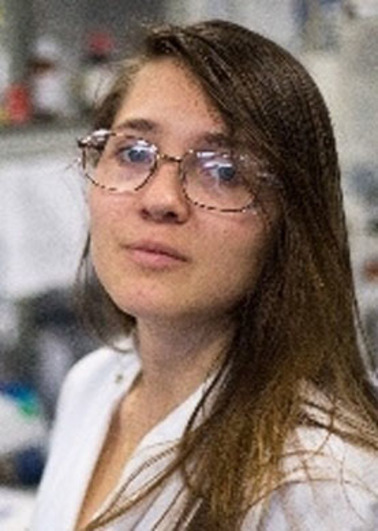

## Biographical Information

*Dr Joe Byrne is Honorary Research Lecturer in the School of Biological and Chemical Sciences, NUI Galway. He currently leads a Science Foundation Ireland Starting Investigator Research Grant project, which includes developing luminescent glycoclusters for lectin sensing. His research interests are in carbohydrate chemistry, coordination chemistry, luminescent systems and supramolecular chemistry. He received his PhD from Trinity College Dublin (2015) and was a Marie Curie Individual Fellow in Universität Bern (2017–19)*.

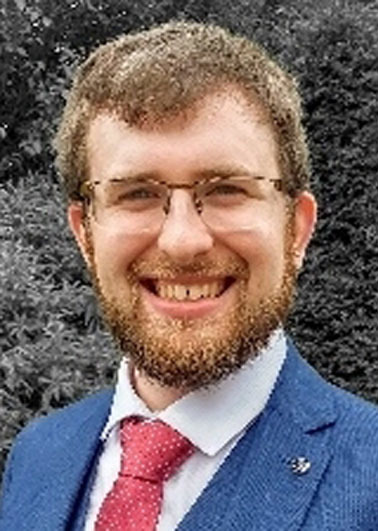
